# Relative Interacting Atomic Energy Analysis of the Anomeric Effect in Cyclic and Acyclic O–C–X Systems

**DOI:** 10.3390/molecules31183335

**Published:** 2026-09-20

**Authors:** Luis R. Domingo, Mar Ríos-Gutiérrez, Patricia Pérez

**Affiliations:** 1 Independent Researcher, Av. Tirso de Molina 20, 46015 Valencia, Spain; 2 Department of Organic Chemistry, University of Valencia, Dr. Moliner 50, 46100 Burjassot, Spain; m.mar.rios@uv.es; 3 Facultad de Ciencias, Escuela de Química y Farmacia, Universidad San Sebastián, Campus Ciudad Universitaria, Av. del Cóndor 720, Ciudad Empresarial, Huechuraba, Santiago 8580704, Chile

**Keywords:** anomeric effect, molecular electron density theory, relative interacting atomic energy, atomic interactions, electron localization function

## Abstract

The conformational preferences of 2-substituted cyclic and acyclic ethers, arising from the *anomeric effect*, have been analyzed using the Relative Interacting Atomic Energy (RIAE) analysis within Molecular Electron Density Theory framework, using the M06-2X/6-311G(d,p) level. Comparison of the equatorial-axial energy differences of substituted tetrahydropyrans and cyclohexanes enables the proposal of the anomeric τ index, which quantifies the excess axial stabilization of the tetrahydropyrans relative to the corresponding cyclohexane references and provides an operational estimate of the contribution associated with the O–C–X motif. The RIAE analysis reveals two distinct patterns in the distribution of the atomic energy contributions associated with anomeric stabilization, dominated by either intra-atomic or interatomic terms, which correlate with the period of the X atom. Accordingly, the studied compounds can be classified into two groups: Group I, comprising compounds with second-period X elements (X = N, O, and F), in which axial stabilization is dominated by favorable intra-atomic energy contributions within the basin of the anomeric carbon; and Group II, comprising compounds with third- and fourth-period X elements (X = S, Cl, and Br), in which stabilization is primarily associated with favorable interatomic energy contributions between the ether oxygen and the anomeric carbon atoms. This atomic energy-based analysis provides a complementary interpretation of the X-dependent conformational trends and identifies analogous energy contribution patterns in the cyclic and acyclic systems examined.

## 1. Introduction

**Scheme 1:**
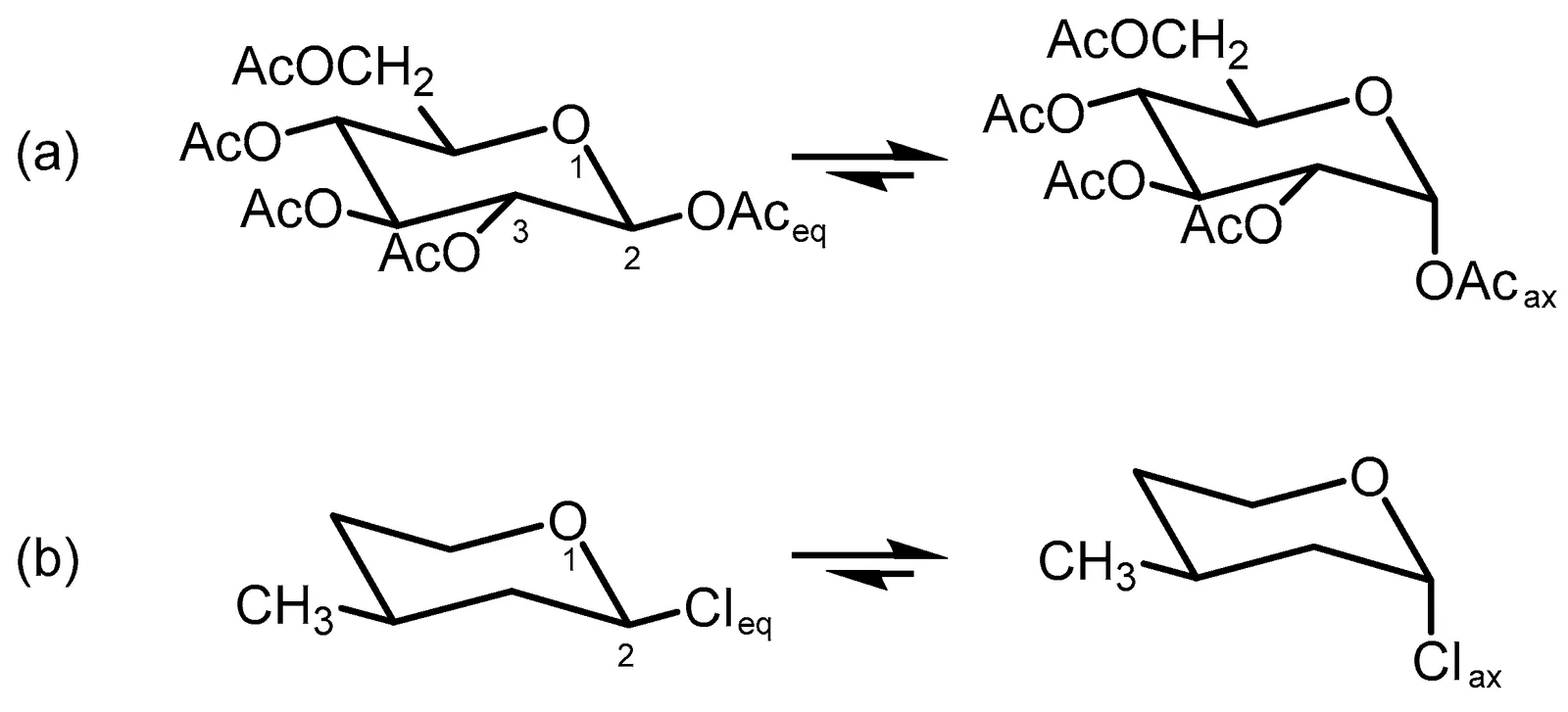
Anomeric effect on 2-substituted (**a**) Pyrs and (**b**) THPs.

The *anomeric effect* is one of the most extensively studied and debated stereoelectronic phenomena in organic chemistry [1]. First recognized in carbohydrate chemistry by Edward [2], and subsequently formalized by Lemieux and Chu [3], it describes the unexpected preference of an electronegative X atom attached to the anomeric carbon for the axial orientation in pyranoses (Pyrs), despite the expected steric preference for the equatorial form (Scheme 1a). This axial stabilization is not restricted to carbohydrates, and it is also observed in simpler heterocycles such as 2-substituted tetrahydropyrans (THPs) (Scheme 1b), and even in acyclic systems containing an O–C–X framework [1,4,5,6,7,8]. The magnitude of the *anomeric effect* is strongly dependent on the nature of the substituent X, with halogens and alkoxy groups often exhibiting markedly different axial–equatorial preferences [1].

**Figure 1:**
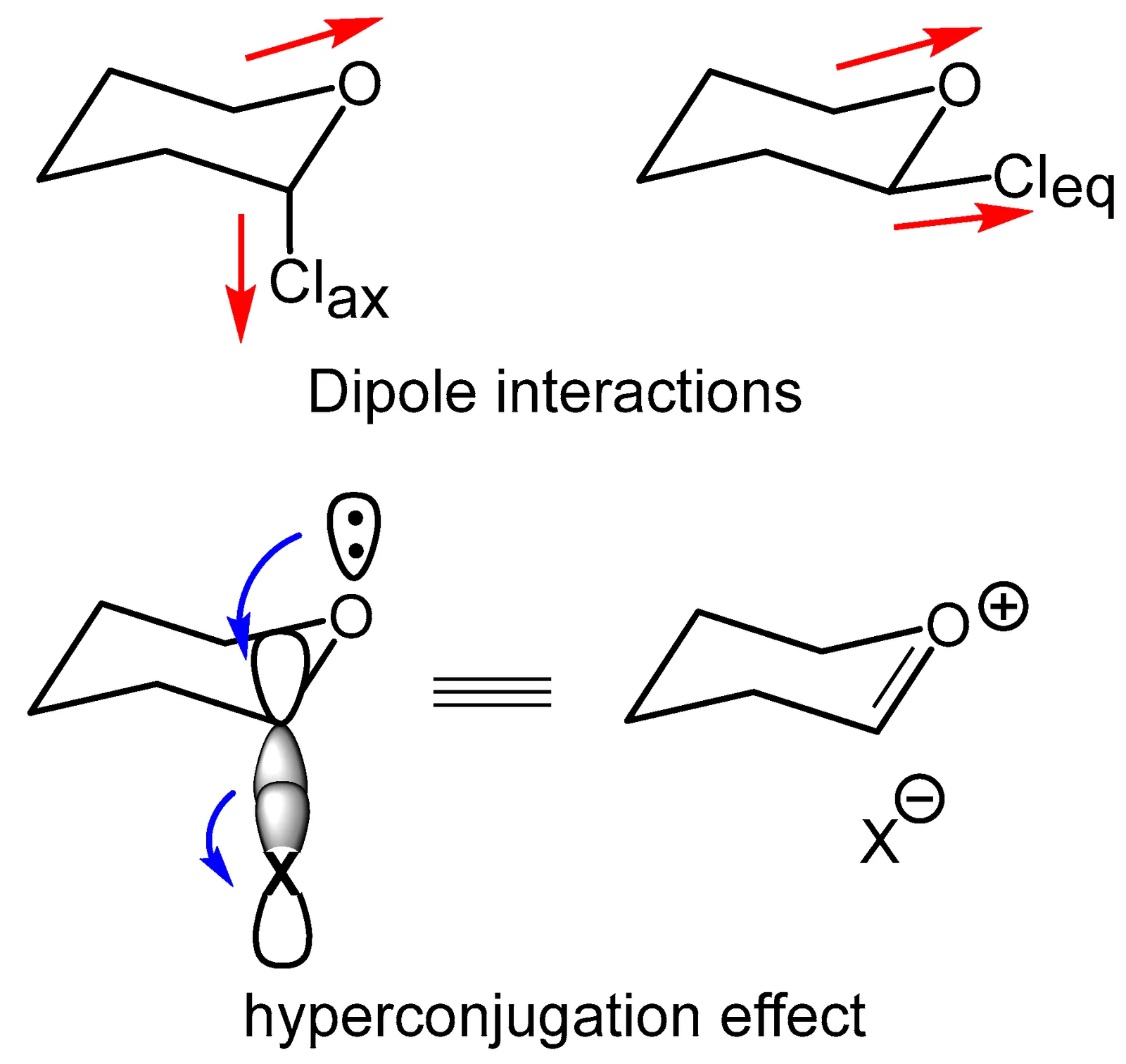
Dipole interactions and hyperconjugation effect proposed in the *anomeric effect*. The bond dipole moments are marked in red, and the proposed electron delocalization in blue.

Two main models have traditionally been proposed to rationalize the *anomeric effect*. The electrostatic model attributed axial stabilization to reducing dipole–dipole repulsion between the non-bonding electron densities of the endocyclic (O) and exocyclic (Cl) heteroatoms in the equatorial form, thereby favoring the axial conformation (see Figure 1) [8]. This approach successfully explained solvent effects, as polar solvents tend to stabilize the equatorial anomer. However, it does not account for the characteristic shortening of the O–C bond and elongation of the C–X bond typically observed in axial conformers [9].

In contrast, the stereoelectronic or hyperconjugative model suggests an interaction between a lone pair on the oxygen (O) and the antibonding (σ*_C–X_) orbital (n_O_ → σ*_C–X_), offering a reason for the geometric features related to the effect and its presence in nonpolar environments (see Figure 1) [9,10,11].

Over the past two decades, more sophisticated theoretical treatments have deepened this debate. Mo’s block-localized wavefunction analysis demonstrated that hyperconjugation contributes far less than previously assumed, and that electrostatic and steric interactions play a dominant role [12]. However, these classical models capture only fragments of the underlying electronic behavior.

**Figure 2:**
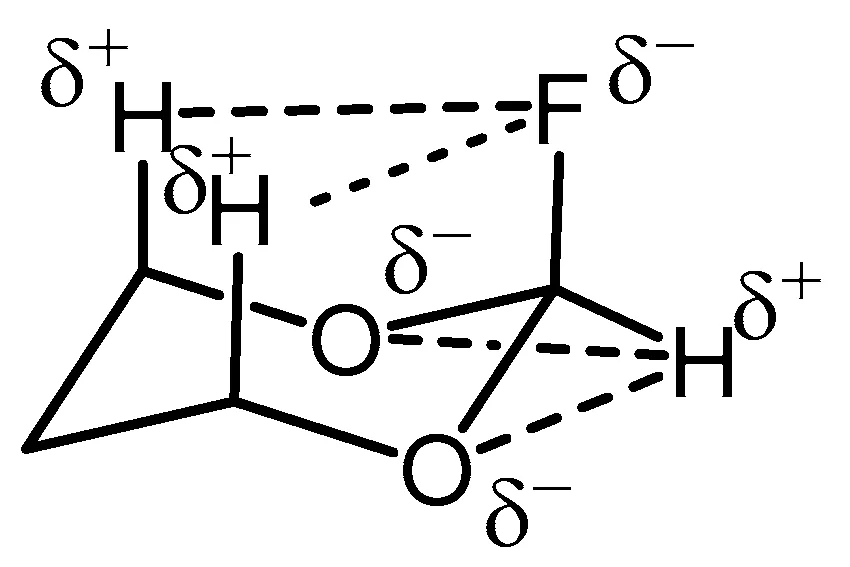
Two major Coulombic attractions responsible for the *anomeric effect* in 2-substituted-1,3-dioxanes.

The conformational preferences of two model compounds of the O–CH_2_–O anomeric unit were analyzed within the framework of the Quantum Theory of Atoms in Molecules [13,14] (QTAIM), finding that the stabilization of gauche conformers was accompanied by a progressive reduction in the electron population of the central methylene hydrogens as the number of gauche interactions with lone pairs increases, resulting in more negative molecular energy [15]. In 2-substituted 1,3-dioxanes, Wiberg et al. suggested that the *anomeric effect* is largely governed by two CH⋯X heteroatom Coulombic attractions rather than by hyperconjugation (Figure 2) [16].

Complementary, energy decomposition approaches emphasized the importance of exchange [17], as well as cooperative contributions from steric, electrostatic, and exchange-correlation terms [18]. Additional methods, such as Interacting Quantum Atoms [19] (IQA) and Relative Energy Gradient [20] (REG) analyses, have further explored these contributions, providing orbital-free views on how electrostatic, exchange, and kinetic factors combine to influence conformational bias. Recently, Popelier et al. [21] used IQA and REG at the B3LYP/6-311++G(d,p) level on both dimethoxymethane and 2-fluorotetrahydropyran, observing an electronic charge shift along the higher-to-lower-energy pathways stabilized by the *anomeric effect*. This redistribution enhances intra-atomic stabilization at the anomeric carbon and increases Coulombic attraction between hydrogens and heteroatoms, indicating that these two energetic contributions are primarily responsible for the effect in both molecules.

Very recently, a multivariate analysis combining computational modeling and statistical methods to assess the origin of the *anomeric effect* has been reported [22]. On the basis of linear regression analyses, the authors concluded that the observed conformational preferences arise from the interplay of several factors, the most significant being (i) stereoelectronic hyperconjugation interactions between the lone pair oxygen and the exocyclic σ* acceptor orbital; (ii) pyramidalization at the anomeric carbon; (iii) steric effects; and (iv) variations in the total molecular surface area associated with dispersion interactions.

Despite these extensive efforts, a fundamental question remains unresolved: whether the *anomeric effect* originates from a single universal electronic mechanism or from distinct stabilization patterns that depend on the electronic nature of the X atom attached to the anomeric carbon [1,12,13,14,15,16,17,18,19,20]. Existing models, based on hyperconjugation, electrostatics, dipole minimization, or CH⋯X interactions, provide partial rationalizations but do not identify which atomic-level interactions are responsible for conformational preferences across different substituent families, nor do they fully explain why similar trends are observed in both cyclic and acyclic systems.

The Molecular Electron Density Theory [23] (MEDT) was proposed in 2016 to investigate chemical processes by studying changes in electron density rather than molecular orbital interactions. MEDT focuses on analyzing changes in electron density along a reaction path and the associated energy costs. Recently, the Relative Interacting Atomic Energy (RIAE) scheme [24,25], an energy decomposition analysis based on the IQA [19] partitioning of Kohn–Sham [26] (KS) Density Functional Theory [27] (DFT) energies, has enabled the analysis of intra- and interatomic energy contributions associated with these changes. Within MEDT, RIAE has proven to be a useful tool for studying the activation energies of relevant organic reactions, including Diels–Alder reactions, [3+2] cycloaddition reactions, and nucleophilic substitution reactions.

**Scheme 2:**
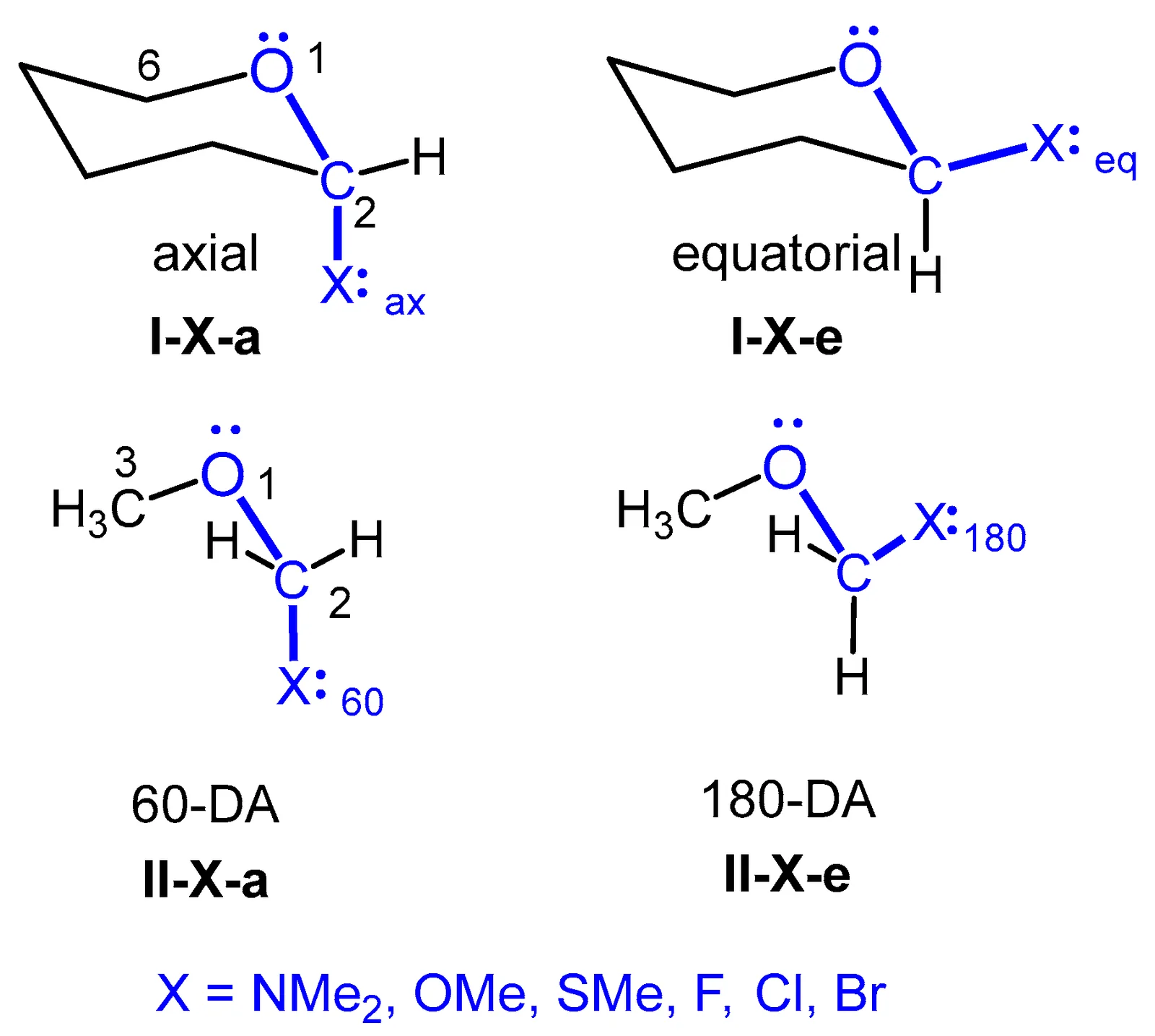
Two axial/equatorial conformations of six cyclic 2-substituted THPs **I**–**X** and the 60-/180-DA conformations of six acyclic substituted MMs **II**–**X**. The O–C–X motif involved in the *anomeric effect* is marked in blue.

The present study addresses the *anomeric effect* within the framework of the MEDT by analyzing two representative families of 2-substituted ethers (Scheme 2): (a) six 2-substituted THPs (**I**–**X**) as cyclic models, in their axial (**I**–**X**–**a**) and equatorial (**I**–**X**–**e**) conformations; and (b) six substituted methoxymethanes or MMs (**II**–**X**) as acyclic models in their 60-degree angle (60-DA; **II**–**X**–**a**) and 180-degree angle (180-DA; **II**–**X**–**e**) conformations, in both families with **X** = **N**, **O**, **F**, **S**, **Cl** and **Br**. These selected molecules contain the O–C–X motif found in Pyrs and substituted THPs, in which this motif is associated with the *anomeric effect* (see Scheme 1). RIAE [24] analyses are used herein to identify the atomic-level electronic factors governing the *anomeric effect*. The relative intra- and interatomic energies [24,25] between the axial/60-DA and equatorial/180-DA conformations are analyzed to characterize the atomic-level energy changes responsible for the observed axial preference in Pyrs and THPs (see Scheme 1).

## 2. Results and Discussion

To elucidate the microscopic origin of the *anomeric effect* in both cyclic and acyclic systems, the relative conformational stabilities of six 2-substituted THPs **I**–**X** and six substituted MMs **II**–**X** are first examined at the M06-2X/6-311G(d,p) level, considering their axial/equatorial or 60-/180-DA conformations. The contribution of the *anomeric effect*, caused by the O–C–X motif, to the axial stabilization of the THP **I**–**X** series, is evaluated by comparison with the substituted cyclohexanes (Chs) **III**–**X**, which lack this structural feature. Solvent effects and thermodynamic calculations for the axial/equatorial stereoisomers of 2-methoxy THP **I**–**O** are performed. Finally, an RIAE analysis of the THPs **I**–**X** and MMs **II**–**X** series is carried out to identify the intra- and interatomic electronic interactions responsible at the atomic level for anomeric stabilization in each molecular family. This combined approach allows us to assess whether the *anomeric effect* arises from a single electronic behavior or from distinct stabilization patterns that depend on the electronic structure of the X atom attached to the anomeric C2 carbon.

Molecules such as Pyrs and THPs can exist in several stereoisomeric forms, each with different three-dimensional molecular geometries and total electronic energies. The corresponding geometries and their associated energies result from different interatomic electronic interactions. A detailed dissection of the IQA $E_{inter}^{AB}$ interatomic energy contributions associated with these interactions is shown in Section S1 of the Supplementary Materials.

### 2.1. Energy Analysis of the Axial and Equatorial Conformations of Cyclic 2-Substituted THPs **I**–**X**

A reliable interpretation of the *anomeric effect* requires first establishing the relative conformational energies, as any electronic behavior must ultimately account for the axial/equatorial stability differences. Therefore, the present study begins by establishing the energetic landscapes of both the cyclic THP and acyclic MM models, which provide the baseline that all geometric, charge, Electron Localization Function (ELF), and RIAE analyses should explain. Due to the great similarities between the axial/equatorial and 60-/180-DA stability gaps and geometrical parameters of both cyclic THP and acyclic MM models, the complete analysis of the acyclic MMs **II**–**X** is presented in Sections S2–S4 of the Supplementary Materials.

Importantly, the axial preference observed in 2-substituted Pyrs and THPs (Scheme 1) does not arise exclusively from the *anomeric effect* associated with the O–C–X motif, but also from additional interatomic electronic interactions involving the atoms belonging to the substituent and the atoms belonging to the cyclic framework. These interatomic electronic interactions can significantly influence the overall conformational stability. For this reason, the 2-substituted THPs **I**–**X** considered in this study were selected to minimize such additional interactions, providing a simplified framework in which the contribution of the O–C–X motif to the *anomeric effect* can be more clearly evaluated.

The axial and equatorial conformations of the six 2-substituted THPs **I**–**X** were optimized at the M06-2X/6-311G(d,p) level in the gas phase. The corresponding axial/equatorial relative energies, Δ$E_{e-a}^{THP}$, are summarized in Table 1, and the total energies of both conformations are provided in Table S9 of the Supplementary Materials. For comparison, the 60- and 180-DA relative energies of the acyclic substituted MMs **II**–**X** (see Δ$E_{e-a}^{MM}$ in Section S2 in the Supplementary Materials) are also included in Table 1.

For the THP series, the axial conformers are consistently more stable than the equatorial ones, with stabilization energies ranging from 2.28 (**I**–**S**) to 4.94 (**I**–**Br**) kcal·mol^−1^. For the halogen series (X = F, Cl, and Br), the axial stabilization increases in the order of 3.23 (F) < 4.26 (Cl) < 4.94 (Br) kcal·mol^−1^. This trend parallels the progression of X element from the second to the fourth period. This trend is reproduced identically in cyclic **I**–**X** and acyclic MMs **II**–**X** (see Section S2 of the Supplementary Materials), indicating that the electronic effects underlying the *anomeric effect* are associated with the O–C–X motif rather than with cyclic constraints.

The axial preferences obtained at the M06-2X/6-311G(d,p) level were compared with those from B3LYP/6-311G(d,p) and M06-2X/6-311++G(d,p) levels to assess the sensitivity of the calculated conformational energies to the chosen computational levels. The corresponding comparative energy analysis is included in Section S5 of the Supplementary Material.

**Scheme 3:**
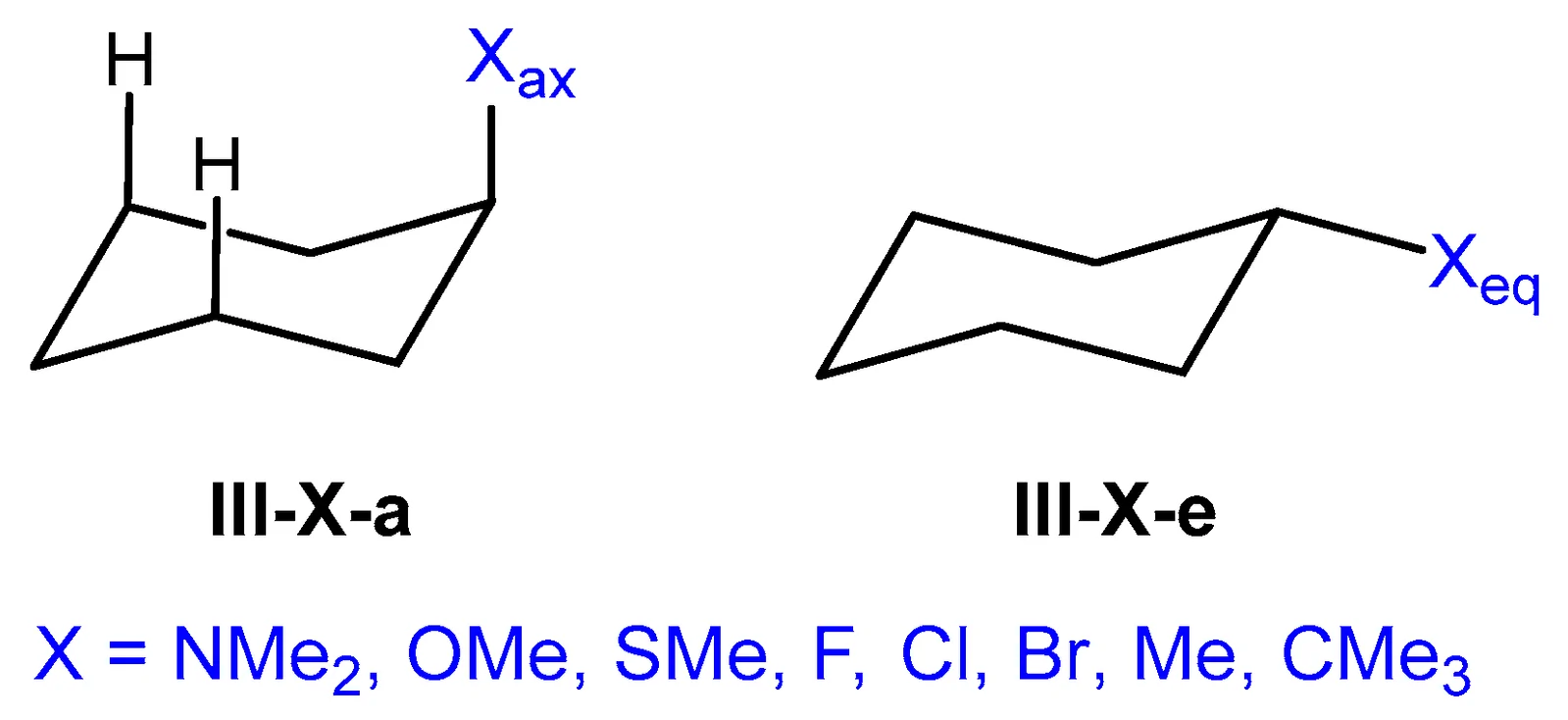
Axial **III**–**X**–**a** and equatorial **III**–**X**–**e** conformations of substituted Chs **III**–**X**.

Importantly, the axial preference observed in 2-substituted Pyrs and THPs (Scheme 1) does not arise exclusively from the *anomeric effect* associated with the O–C–X motif, but also from additional electronic interactions involving the atoms in the substituent and those in the cyclic framework. To minimize these secondary contributions, the present THP **I**–**X** series was selected as a simplified model system of 2-substituted Pyrs. Nevertheless, residual electronic effects unrelated to the O–C–X motif may still contribute to the conformational preferences. To assess these contributions, the axial and equatorial conformations of eight substituted cyclohexanes, Chs, **III**–**X**, which lack the O–C–X motif (see Scheme 3), were also optimized. The corresponding relative energies Δ$E_{e-a}^{Ch}$ are included in Table 1, and total energies are reported in Table S10 of the Supplementary Materials. The hydrocarbons, methylcyclohexane **III**–**Me** and tert-butylcyclohexane **III**–**tBu**, were also studied. Negative values of ΔE_e−a_ in Table 1 indicate that the equatorial conformation is the most stable.

As expected, steric effects dominate in alkyl-substituted Chs: the axial conformations of methylcyclohexane **III**–**Me**–**a** and tert-butylcyclohexane **III**–**tBu** are 1.50 and 4.55 kcal·mol^−1^, respectively, higher in energy than the equatorial conformations. The conformational Gibbs free energies for Chs **III**–**Me** and **III**–**tBu** have been estimated at 1.8 and 4.7 kcal·mol^−1^, respectively [1], closer to the M06-2X/6-311G(d,p) gas-phase relative energies of the substituted alkyl. Steric 1,3-diaxial interactions between the axial methyl and tert-butyl groups and the two axial hydrogens have been proposed to account for these relative energies in hydrocarbon systems (see **III**–**X**–**a** in Scheme 3) [1]. These unfavorable interactions are also present in the axial 2-methyl THP **I**–**Me**–**a**, which is 2.42 kcal·mol^−1^ higher in energy than the equatorial **I**–**Me**–**e** (see Table 1).

In contrast, the substituted Chs **III**–**X** exhibit X-atom-dependent electronic effects. For this series, the axial conformation of the N, O, and F heteroatoms **III**–**X**–**a** is between 0.42 and 2.56 kcal·mol^−1^ more stable than the equatorial conformation **III**–**X**–**e**. For the S atom, **III**–**S**, the axial conformation is 0.24 kcal·mol^−1^ less stable than the equatorial one, while for Cl and Br atoms, **III**–**Cl** and **III**–**Br**, both conformations have similar energies (see Table 1). These results suggest that the X atom-dependent electronic interactions within the cyclohexane framework, which are also present in the 2-substituted THPs **I**–**X**, vary significantly with the nature of the X element. Notably, Chs **III**–**O** and **III**–**S** exhibit opposite conformational preferences, with the former favoring the axial conformation and the latter the equatorial one.

Consequently, the axial/equatorial energy differences observed for THPs **I**–**X** (see Δ$E_{e-a}^{THP}$ in Table 1) reflect not only the so-called *anomeric effect* associated with the electronic interaction at the O–C–X motif, but also additional electronic interactions, mainly between atoms belonging to the group attached to the anomeric C2 carbon and those of the cyclic hydrocarbon chain, which are also present in substituted Chs **III**–**X**.

The axial/equatorial energy differences observed between the THP **I**–**X** series, Δ$E_{e-a}^{THP}$, and those observed in the Ch **III**–**X** series, Δ$E_{e-a}^{Ch}$, allow for an estimation of the *anomeric effect* caused by the X atom of the O–C–X motif present in the THP series. The corresponding energy difference permits the definition of the anomérico τ index as:

(1) $$\tau \;=\;\Delta E_{e-a}^{THP}\;-\;\Delta E_{e-a}^{Ch}$$

i.e., the difference between the equatorial/axial relative energies of the C2-substituted THPs and the corresponding substituted Chs. The sign convention adopted here is $\Delta E_{e-a}=E_{e}-E_{a}$; therefore, positive values indicate an axial preference. The anomeric τ  index is defined as an operational, reference-based energy descriptor of the excess axial stabilization of THP **I**–**X** relative to the corresponding substituted cyclohexane Ch **III**–**X**. This comparison assumes that substituent-dependent interactions with the hydrocarbon framework that are present in both systems are transferable to the first order and are therefore partially canceled by the subtraction. Because replacing the ring oxygen of THP with a methylene group can also modify molecular geometry and polarization, this cancelation need not be exact. Accordingly, the τ  index should be regarded as an estimate of the conformational stabilization associated with the O–C–X motif rather than as a rigorous energy decomposition or a directly observable quantity. The resulting M06-2X/6-311G(d,p) τ values for the six 2-substituted THPs **I**–**X** are reported in Table 1. The positive τ values in Table 1 reflect a stabilizing *anomeric effect* caused by the X atom, which favors the axial conformation.

**Figure 3:**
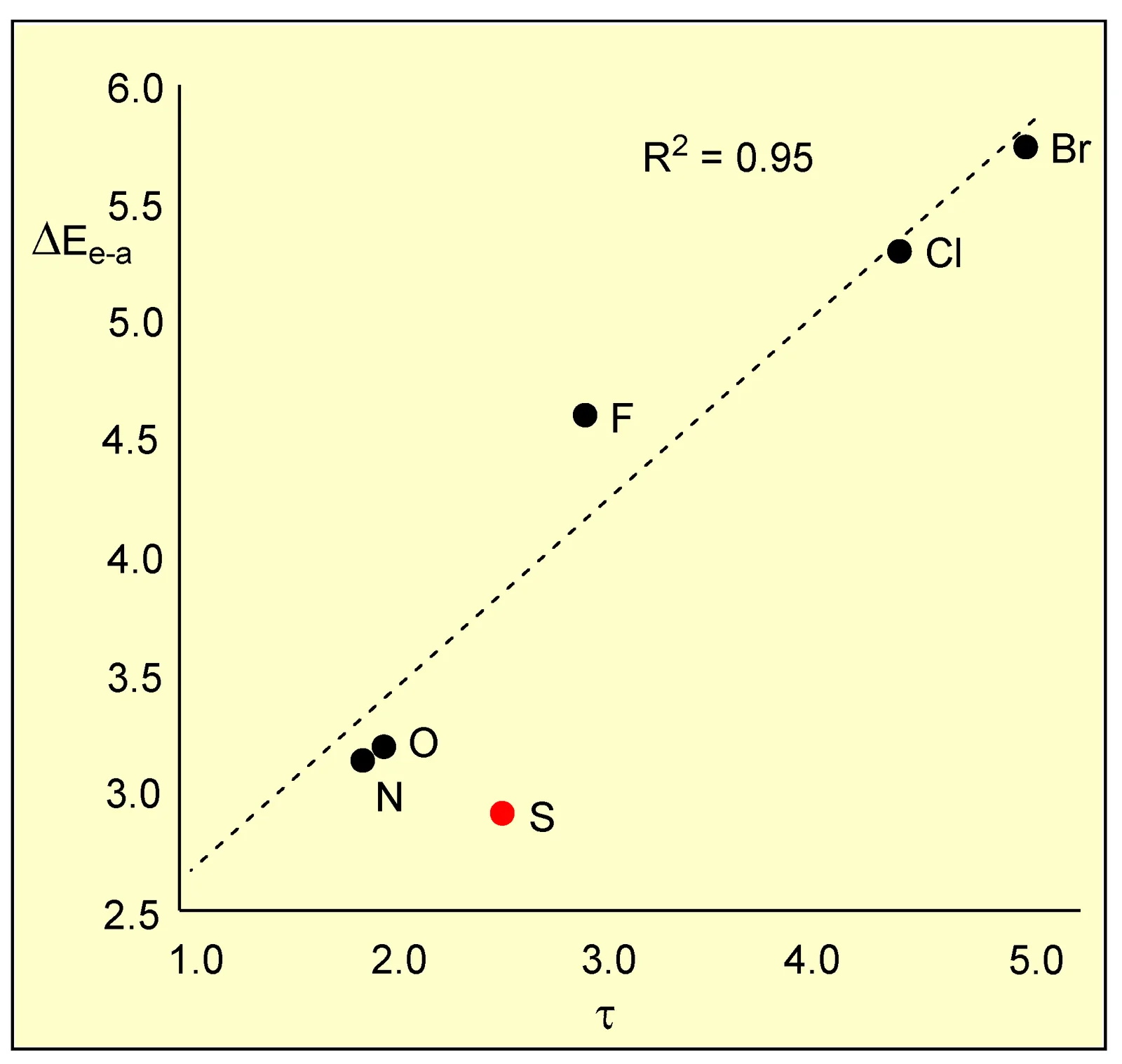
Plot of the M06-2X/6-311G(d,p) gas-phase relative energies, ΔE_e−a_, between the corresponding 60- and 180-DA conformations of acyclic substituted MMs **II**–**X** versus the corresponding anomeric τ index (see Table 1). Energies are given in kcal·mol^−1^. MM **II**–**S**, which is shown in red, was excluded from the linear regression.

Figure 3 shows the relationship between the axial preference of the acyclic MMs **II**–**X** series, which minimizes interatomic electronic interactions not associated with the O–C–X motif present in THPs **I**–**X**, and the corresponding anomeric τ values obtained from Equation 1. MM **II**–**S** deviates markedly from the trend observed in the other systems (the red point in Figure 3). Excluding MM **II**–**S** from an exploratory linear regression yields a strong correlation for the remaining five compounds (R^2^ = 0.95, see Figure 3). This result suggests that, with the notable exception of the sulfur derivative, the proposed τ index captures the variation in the conformational stabilization associated with the 60 DA arrangement of the O–C–X motif in the acyclic MM **II**–**X** series.

**Figure 4:**
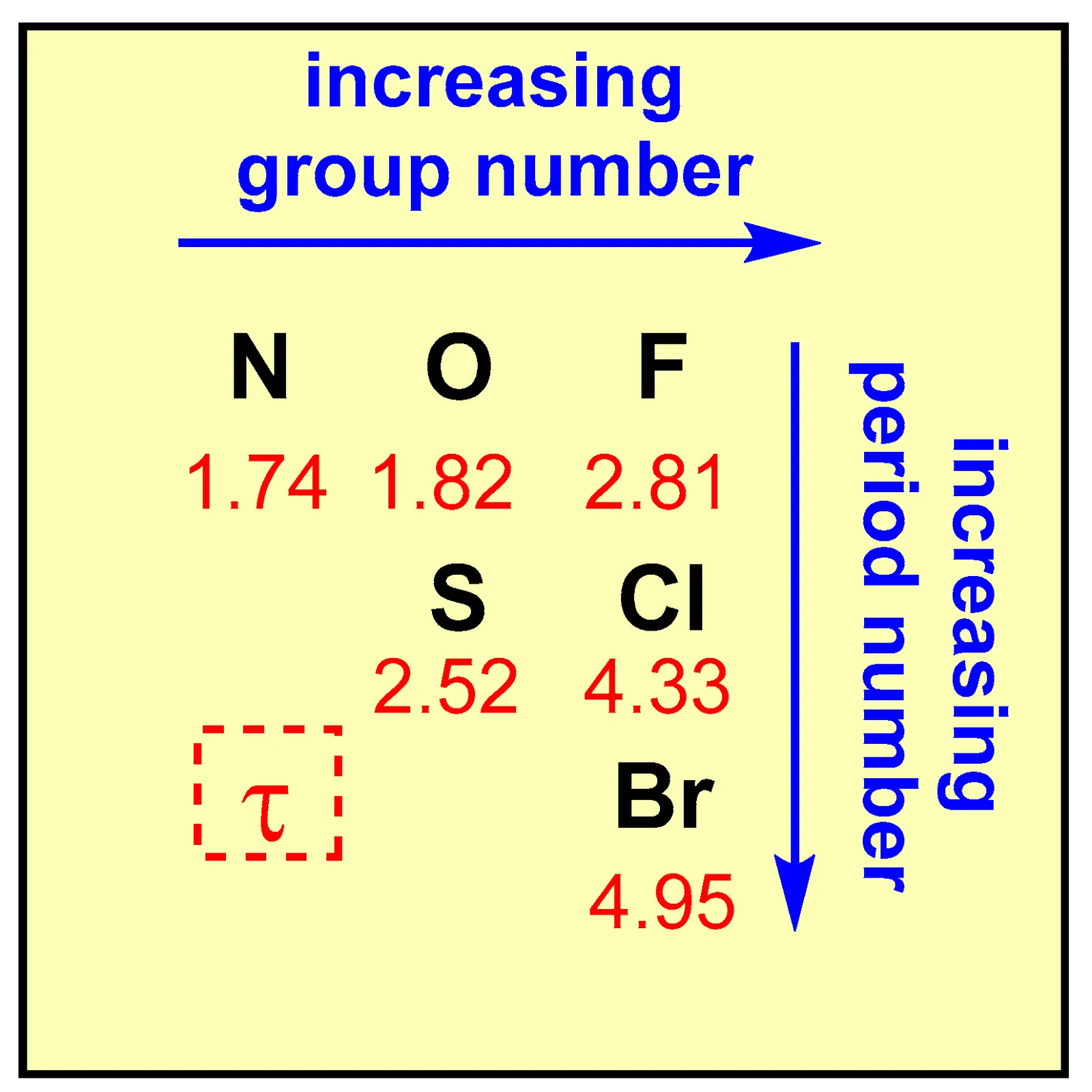
Schematic representation of the proposed anomeric τ indices computed at the M06-2X/6-311G(d,p) level for the 2-substituted THPs **I**–**X**, arranged according to the group and period of the X atom in the periodic table. The τ values are given in kcal·mol^−1^.

Figure 4 shows a schematic representation of the proposed anomeric τ indices computed at the M06-2X/6-311G(d,p) level for the 2-substituted THPs **I**–**X** arranged according to the group and period of the X atom in the periodic table. Analysis of the M06-2X/6-311G(d,p) τ values reveals three main trends: (i) the anomeric τ index increases in the order of 1.74 (N) < 1.82 (O) < 2.52 (S) < 2.81 (F) < 4.33 (Cl) < 4.95 (Br) kcal·mol^−1^ (see Figure 4), showing a systematic dependence on the nature of the X element; (ii) within the second-period series, τ increases in the order of 1.74 (N) < 1.82 (O) < 2.81 (F) kcal·mol^−1^, paralleling the increase in Pauling electronegativity, χ = 3.04 (N) < 3.44 (O) < 3.98 (F); and (iii) within the halogen series τ increases in the order of 2.81 (F) < 4.33 (Cl) < 4.95 (Br) kcal·mol^−1^, as X element progresses from the second to the fourth period, whereas electronegativity decreases in the opposite direction, χ = 3.98 (F) > 3.16 (Cl) > 2.96 (Br) [1]. Note that this increase is also observed in the short series of the chalcogen group τ = 1.82 (O) < 2.52 (S). Thus, for the halogen derivatives, the increase in τ correlates with the X element period rather than with its electronegativity. The largest τ value, 4.95 kcal·mol^−1^, is found for the bromine derivative **I**–**Br**–**a**, whereas the smallest value, 1.74 kcal·mol^−1^, is obtained for the nitrogen derivative **I**–**N**–**a**.

Interestingly, the strong axial stabilization of the ether-substituted Ch **III**–**O**, Δ$E_{e-a}^{Ch}$ = 2.56 kcal·mol^−1^, accounts for the strong stabilization of THP **I**–**O**, Δ$E_{e-a}^{THP}$ = 4.38 kcal·mol^−1^ (see Table 1). Thus, except for THP **I**–**O**, where the intrinsic stabilization of the axial conformation exceeds the τ value of 1.82 kcal·mol^−1^, the τ index makes a major contribution to axial stabilization in the remaining THPs, indicating that the anomeric contribution dominates other electronic interactions. For comparison, the corresponding 60-DA stabilization of MMs **II**–**O** is 3.19 kcal·mol^−1^ (see Table 1). Therefore, excluding the electronic interactions present in the cyclohexane framework of the Ch **III**–**X** series from those of the THP **I**–**X** series allows the anomeric τ index to be estimated. These values can be used as an approximate estimation of the *anomeric effect* caused by the O–C–X motif present in 2-substituted Pyrs and THPs.

The calculated axial preferences show dependence on the computational method, leading to the calculation of corresponding τ indices at the B3LYP/6-311G(d,p) level (see Section S5 of the Supplementary Material). Although their numerical magnitude remains method-dependent, meaning that fine energetic differences should not be overinterpreted, both the B3LYP and M06-2X methods reproduce the general variation of τ across the series and exhibit the same upward trend with increasing group and period numbers. An excellent linear correlation is found between the τ values obtained with the M06-2X and B3LYP functionals, with R^2^ = 1.00.

### 2.2. Solvent Effects and Thermodynamic Calculations for the Axial/Equatorial Stereoisomers of 2-Methoxy THP **I**–**Oa**

Solvent effects on the *anomeric effect* were originally examined by Lemieux et al. [28], who showed that the population of the axial conformer in 2-methoxy THP decreases as solvent polarity increases. To analyze this behavior, the axial/equatorial conformational equilibrium of 2-methoxy THP **I**–**O** was studied in a series of solvents with an increasing dielectric constant.

**Figure 5:**
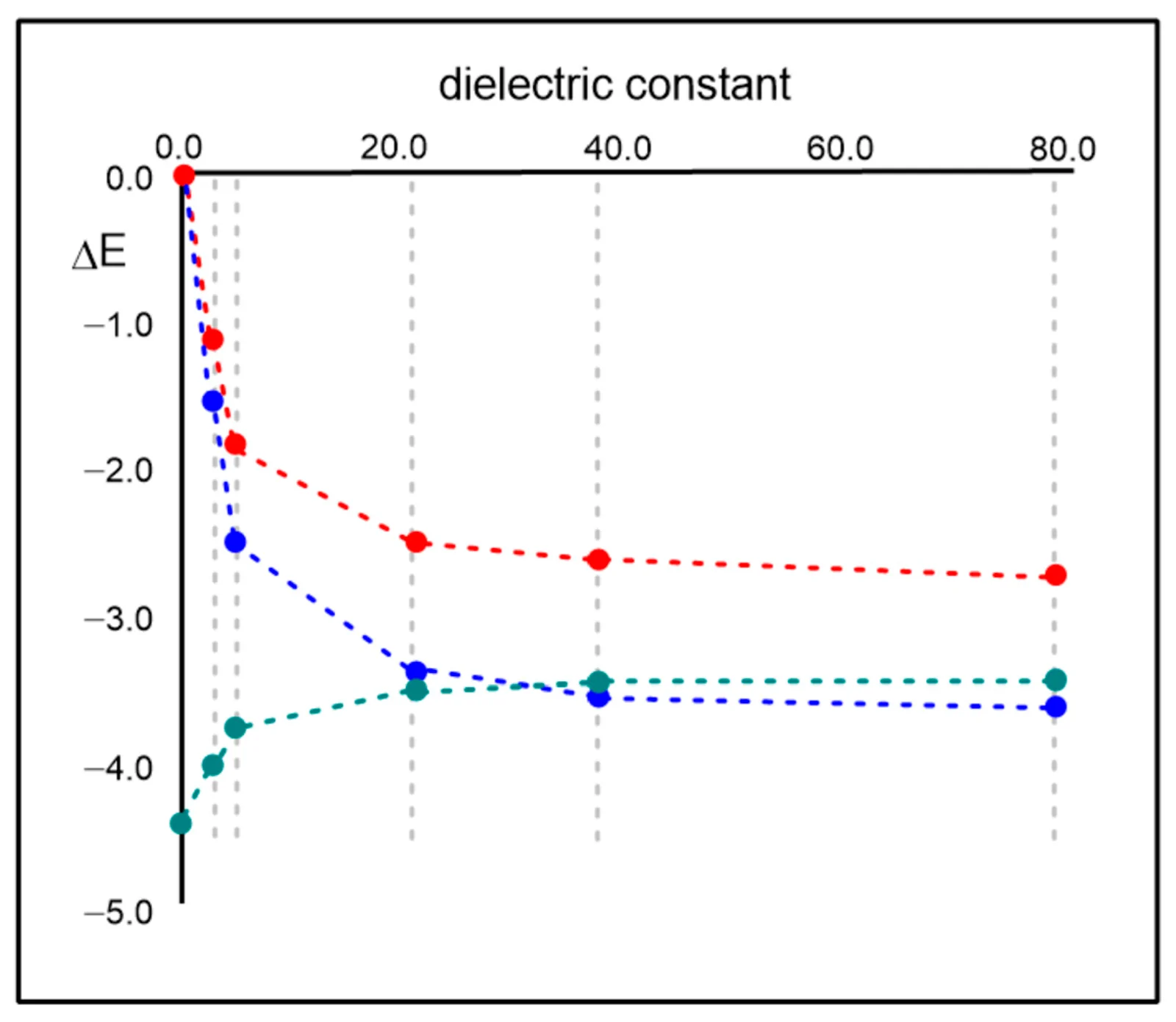
Graphical representation of the Δ$E_{ax}^{s}$, in red, Δ$E_{eq}^{s}$, in blue, and Δ$E_{ae}^{s}$, in green, relative energies given in Table 2, in kcal·mol^−1^, with respect to the dielectric constant ε.

The M06-2X/6-311G(d,p) relative solvation energies of the axial (Δ$E_{ax}^{s}$) and equatorial (Δ$E_{eq}^{s}$) conformations of THP **I**–**O**, together with the corresponding axial-equatorial energy differences in the selected solvents Δ$E_{ae}^{s}$ are summarized in Table 2 and represented in Figure 5.

Three main observations arise from Figure 5: (i) first, the equatorial conformer is increasingly stabilized relative to the axial one as the solvent dielectric constant increases. This behavior is consistent with the larger dipole moment of the equatorial stereoisomer, THP **I**–**O**–**e**, relative to the axial one, THP **I**–**O**–**a**, 2.13 and 0.35 Debye, respectively. (ii) Second, although both conformers are stabilized upon solvation, the magnitude of this stabilization becomes nearly constant for dielectric constants above ~20. (iii) Third, despite the preferential stabilization of the equatorial conformer, the axial–equatorial energy difference (Δ$E_{ae}^{s}$) remains nearly unchanged at higher dielectric constants. This behavior suggests that the electronic interactions responsible for the *anomeric effect* are only weakly modulated by solvation. These results indicate that, although polar solvents decrease the axial–equatorial energy difference through the greater solvation of the more polar and less stable equatorial conformation, the overall magnitude of the *anomeric effect* remains only weakly affected by the solvent environment.

The axial/equatorial relationships of 2-substituted Pyrs or THPs (given in Scheme 1) are experimentally determined by spectroscopic techniques such as NMR, which provide the composition of the stereoisomeric mixture. From these data, the Gibbs free energy differences between the axial/equatorial stereoisomers can be determined. To correlate the computed relative axial/equatorial electronic energies Δ$E_{ae}^{s}$ with the relative Gibbs free energies experimentally obtained, the thermodynamic calculations of the axial/equatorial equilibrium for 2-methoxy THP **I**–**O** in three selected solvents of increasing polarity were performed. The relative axial/equatorial electronic energies, enthalpies, and Gibbs free energies Δ$E_{ae}^{s}$, Δ$H_{ae}^{s}$, and Δ$G_{ae}^{s}$ in kcal·mol^−1^, and entropies Δ$S_{ae}^{s}$ in cal·mol^−1^·K^−1^, computed in the corresponding solvent at room temperature, are given in Table 3.

Two main conclusions can be obtained from Table 3: (i) The relative axial/equatorial Gibbs free energies for the equilibrium of THP **I**–**O** are only 0.07 kcal·mol^−1^ less than the relative electronic energies. This behavior is a consequence of two factors: (a) the variation between the relative electronic energies Δ$E_{ae}^{s}$ and enthalpies Δ$H_{ae}^{s}$ are less than 0.1 kcal·mol^−1^; and (b) the variation in entropies between the axial and equatorial conformations is negligible (${∆S}_{ae}^{s}$ < 0.28 cal·mol^−1^·K^−1^). (ii) The axial/equatorial relationships experimentally determined can be correlated with the inter- and intra-atomic electronic interactions present in the axial/equatorial stereoisomer, which include the so-called *anomeric effects*.

### 2.3. Geometrical and Atomic Charge Analyses of Cyclic 2-Substituted THPs **I**–**X**

**Figure 6:**
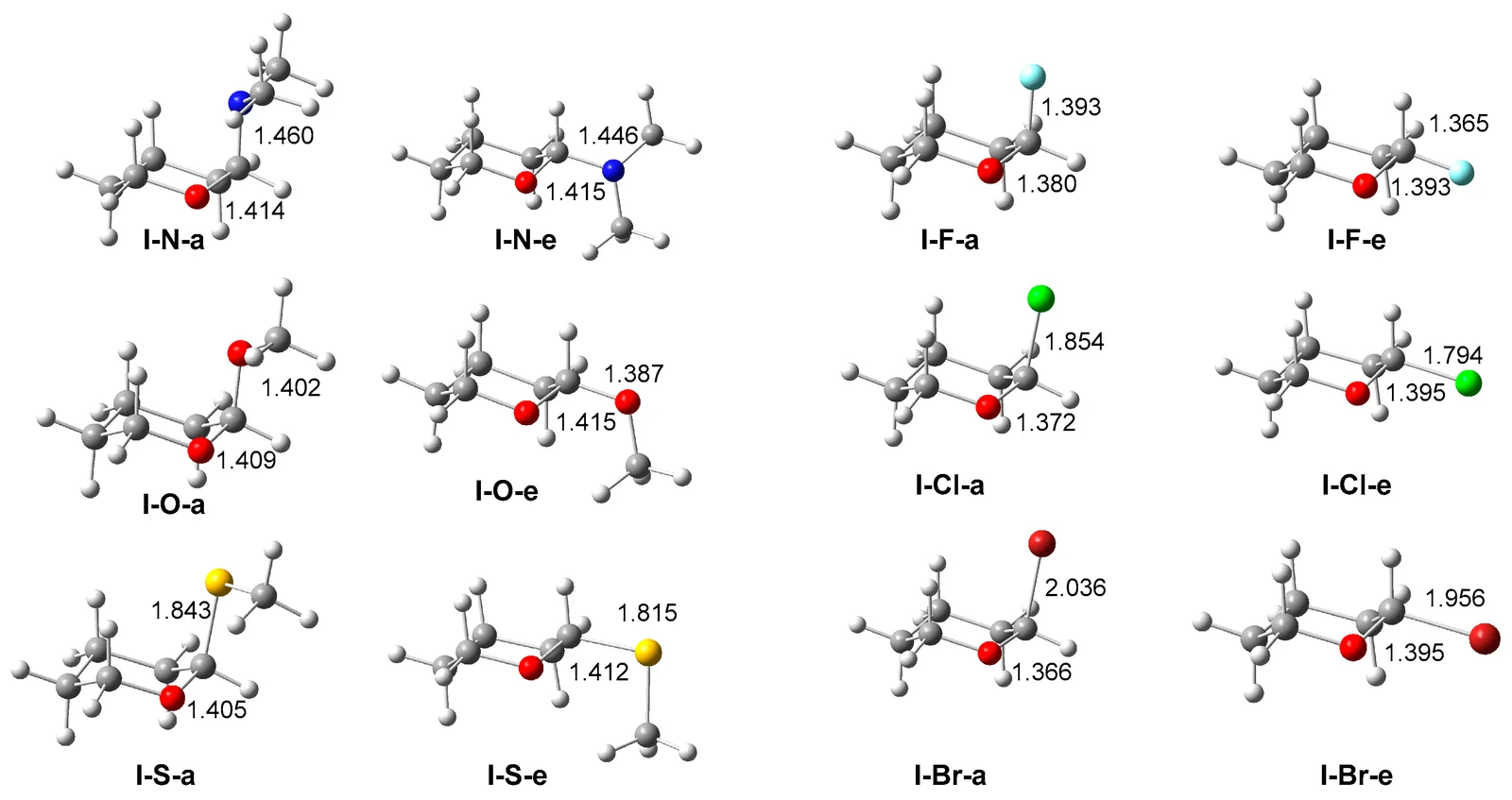
M06-2X/6-311G(d,p) gas-phase optimized geometries of axial and equatorial conformations of THPs **I**–**X**. The bond lengths are given in angstroms (Å).

We next asked whether geometric parameters and the natural atomic charges of the O1–C2–X framework in the axial and equatorial conformations of cyclic 2-substituted THPs **I**–**X** correlate with the energetic trends observed in Section 2.1. The optimized geometries of substituted THPs **I**–**X** are given in Figure 6. The differences in the O1–C2 and C2–X bond lengths between the two conformations, Δl_a−e_, are summarized in Table 4, while natural atomic charges of the O1–C2–X framework are reported in Table 5. The corresponding parameters for the acyclic MMs **II**–**X** series are given in Section S3 of the Supplementary Materials.

Analysis of the O1–C2 and C2–X bond-length differences (reported in Table 4) reveals several systematic trends: (i) First, the O1–C2 bond is consistently shorter in the axial conformers, with values of Δl_a−e_ ranging from −0.001 to −0.029 Å, whereas the C2–X bond is elongated by 0.015–0.080 Å. The simultaneous O1–C2 contraction and C2–X elongation correspond to the structural pattern conventionally associated with anomeric stabilization. (ii) Second, within the second-period series, the magnitude of these structural changes generally increases in the order of N < O < F, although identical C2–X elongations are obtained for the N and O derivatives. (iii) Within the halogen series, both the O1–C2 contraction and C2–X elongation increase markedly in the order of F < Cl < Br. The sulfur derivative does not follow a simple period-dependent trend; its C2–S elongation is identical to that obtained for C2–F, whereas its O1–C2 contraction is intermediate between those of the O- and F-substituted derivatives. Thus, the observed structural changes depend on the identity of X and cannot be described solely in terms of its period. Finally, analogous O1–C2 contractions and C2–X elongations are obtained for the acyclic MMs **II**–**X** (Section S3 of the Supplementary Materials). These structural variations broadly parallel the corresponding conformational energy trends, although they do not by themselves establish their electronic origin.

The analysis of the atomic charges obtained by natural population analysis [29,30] (NPA) for the three atoms in the O–C–X motif provides additional information on the electronic polarization of the O1–C2–X framework (Table 5). The corresponding atomic charges for the 60- and 180-DA conformations of the substituted MMs **II**–**X** are given in Section S3 of the Supplementary Materials. Several conclusions emerge from Table 5: (i) First, the net charge of the O1 oxygen remains nearly constant (ca. −0.60 e) across both conformations and throughout the substituent series, indicating that this quantity is relatively insensitive to the conformational change. In contrast, larger substituent- and conformation-dependent variations are observed at C2 and the X atoms. (ii) Second, the positive charge at the anomeric C2 carbon is considerably larger for the derivatives containing second-period X elements (X = N, O, and F; 0.34–0.58 e) than for those containing third- and fourth-period X elements (X = S, Cl, and Br; 0.02–0.21 e). Within the second-period series, the positive charge at C2 increases in the order of N < O < F, paralleling the increase in the electronegativity of X. (iii) Third, the C2–X bond is more strongly polarized in the second-period derivatives, for which X bears a substantial negative charge (−0.39 to −0.61 e). By contrast, S bears a positive charge, whereas Cl and Br carry comparatively small negative charges. Finally, (iv) the axial–equatorial charge differences occur predominantly at C2 and X, and are larger for the S-, Cl-, and Br-substituted derivatives, particularly at X. Overall, the charge analysis results identify two distinct substituent-dependent polarization patterns: one for compounds containing second-period X elements and another for those containing third- and fourth-period X elements.

The calculations show that the net NPA charge at O1 oxygen changes only slightly between the axial and equatorial conformations of THPs **I**–**X** (approximately −0.61 e; Table 5) and between the 60-DA and 180-DA conformations of MMs **II**–**X** (approximately −0.58 e; Table S4 of the Supplementary Materials). This indicates that the conformational change produces only a small variation in the net NPA population assigned to O1.

Overall, the relative energies (Table 1), bond-length variations (Table 4), and atomic charge distributions at C2 and X (Table 5) reveal substituent-dependent patterns that correlate with the period and electronegativity of X. Nevertheless, these geometrical and charge descriptors do not by themselves establish the physical origin of the conformational energy trends. The ELF and RIAE analyses presented below, therefore, provide complementary descriptions of the electron-density organization and of the distribution of the atomic energy contributions associated with anomeric stabilization.

### 2.4. ELF Topological Analyses of the 60- and 180-DA Conformations of MMs **II**–**F** and **II**–**Cl**

**Figure 7:**
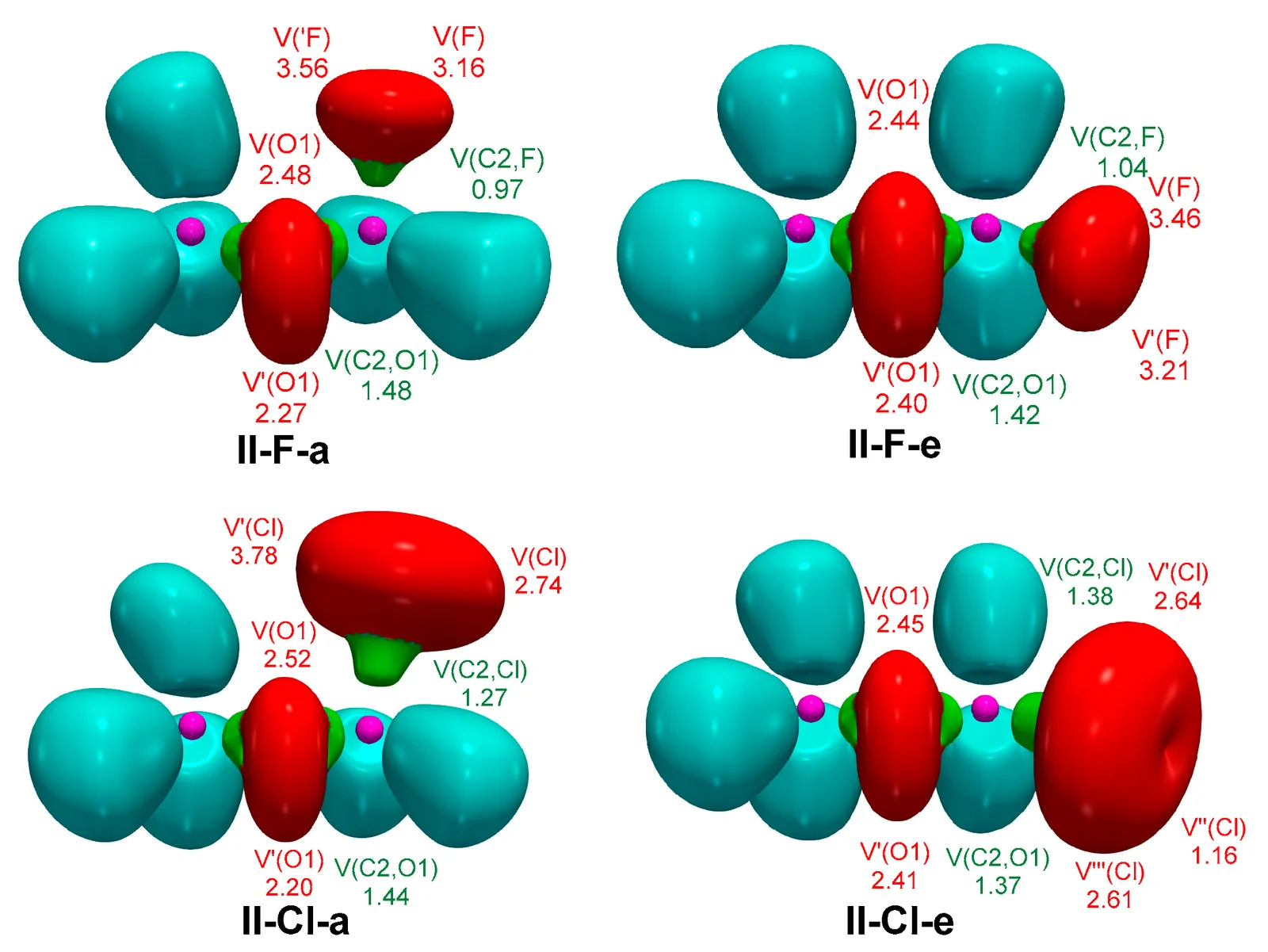
M06-2X/6-311G(d,p) ELF valence basins at the 60- and 180-DA conformations of MMs **II**–**F** and **II**–**Cl**. The populations of the relevant V(C2,O1) and V(C2,X) disynaptic basins, in green, and V(O1) and V(X) monosynaptic basins, in red, are given as the average number of electrons (e).

The topological analysis of ELF [31] provides a quantitative description of the electron density distribution within a molecule [32], offering direct insight into its electronic structure and reactivity. To examine the electron density changes between the axial/60-DA and equatorial/180-DA conformations of THPs **I**–**X** and MMs **II**–**X**, ELF analyses were performed for the 60- and 180-DA conformations of the acyclic MMs **II**–**F** and **II**–**Cl**. These compounds were selected as representative systems containing a second-period X element (F) and a third-period X element (Cl). The use of these acyclic models reduces the topological complexity associated with the cyclic THPs and facilitates comparison of the ELF basin populations directly associated with the O1–C2–X motif. The corresponding ELF valence basin distributions and the most relevant valence basin populations are depicted in Figure 7.

In all four structures, the ELF topology consists of one V(C2,O1) disynaptic basin integrating less than 1.48 e, and one V(C2,X) disynaptic basin integrating less than 1.04 e for X = F or less than 1.38 e for X = Cl. These depopulated disynaptic basins correspond to the polarized C2–O1 and C2–X bonding regions. Each molecule also features two O1-centered monosynaptic basins, V(O1) and V′(O1), integrating less than 4.72 e in total, and two at **II**–**F** or two/three at the **II**–**Cl** monosynaptic V(X) basins, integrating a total of less than 6.67 e and 6.25 e, respectively. These highly populated V(X) monosynaptic basins represent the non-bonding electron density at the O, F, and Cl atoms, which is consistent with their higher electronegativity relative to the bonded C1 carbons.

ELF comparison of the two conformations of **II**–**F** and **II**–**Cl** reveals a similar pattern. In the more stable 60-DA conformer **II**–**F**–**a**, the C2–O1 single bond and the F non-bonding region are slightly more populated, whereas the C2–F single bond is slightly depopulated. In the favored 60-DA conformer **II**–**Cl**–**a**, the C2–O1 single bond and the O and Cl non-bonding regions show a small increase in population, while the C2–Cl single bond is slightly depopulated.

The depopulation of the V(C2,X) disynaptic basins in the 60-DA conformations accounts for the slightly longer C2–X distances observed in axial and 60-DA conformations. On the other hand, the slightly higher populations of the V(C2,O1) disynaptic basins at 60-DA conformations account for the shorter C2–O1 distances in axial and 60-DA conformations (see Figure 6 and Figure S1 of the Supplementary Materials).

Finally, Figure 7 shows that the V(Cl) monosynaptic basins in **II**–**Cl** occupy a larger volume than the V(F) monosynaptic basins in **II**–**F**, consistent with Cl being a third-period element and F a second-period element.

### 2.5. RIAE Analysis of the 2-Substituted THPs **I**–**X**

In order to determine the electronic interactions at an atomic level responsible for the *anomeric effects*, RIAE [24,25] analyses for the 2-substituted THPs **I**–**X** and substituted MMs **II**–**X** were performed. To our knowledge, stereoisomeric energy differences have not previously been examined using RIAE. In this approach, the relative ξ$E_{intra}^{A}$ intra-atomic and ξ$E_{inter}^{AB}$ interatomic energies between the two conformations were computed and analyzed. The relative ξ$E_{total}^{ae}$ total energies obtained by summing the relative ξ$E_{intra}^{A}$ intra-atomic and ξ$E_{inter}^{AB}$ interatomic energies represent the RIAE relative energies between the two stereoisomers. This approach enables the energetic origin of the axial/equatorial stability differences to be examined in terms of atomic contributions, providing a complementary perspective to the structural and electron-density analyses discussed above.

The RIAE analysis is organized in three parts: (i) the six 2-substituted THPs **I**–**X**, (ii) the corresponding MMs **II**–**X**, and (iii) methoxycyclohexane **III**–**O** and methylcyclohexane **III**–**Me**, as reference systems lacking the O–C–X motif. Due to the similarity of the RIAE of MMs **II**–**X** with that of THPs **I**–**X**, the former RIAE analysis is presented in Section S4 of the Supplementary Materials.

#### 2.5.1. RIAE Analysis of 2-Substituted THPs **I**–**X**

First, the RIAE analysis for the six 2-substituted THPs **I**–**X** was performed to identify—at the atomic energy level—the electronic factors responsible for the preferred axial conformation. Taking advantage of the topological atoms [33], Popelier recently proposed an Interacting Quantum Fragment approach [34], which allows grouping the IQA energy terms into chemically meaningful fragments ƒ(X) of the molecular system.

To examine the atomic energy contributions associated with the O–C–X motif, the atoms of THPs **I**–**X**, were grouped into two fragments: (a) the three atoms defining the O–C–X motif, and (b) the remaining hydrocarbon framework, denoted as CH. The M06-2X/6-311G(d,p) axial/equatorial relative ξ$E_{intra}^{X}\;$ intra-atomic, ξ$E_{inter}^{X}$ interatomic, and ξ$E_{total}^{X}$ total energies for the OCX and CH frameworks of THPs **I**–**X** are given in Table 6. The ξ$E_{total}^{ae}$, which corresponds to the total axial/equatorial stabilization obtained by summing the contributions assigned to both fragments, represents the total axial–equatorial conformational energy differences recovered from the RIAE partition. Note that the relative ξ$E_{intra}^{X}$ and ξ$E_{inter}^{X}$ contributions are defined using the axial-minus-equatorial convention, $\xi E_{a-e}=E_{a}-E_{e}$. Accordingly, negative values represent contributions favoring axial stabilization. Consequently, the ξ$E_{total}^{ae}$ values reported in Table 6 follow the opposite sign convention to that used for the anomeric τ index in Table 1, for which positive values indicate an excess axial stabilization.

Importantly, $\xi E_{total}^{OCX}\;$ should not be equated with the anomeric τ index. The τ index is a reference-based conformational energy descriptor obtained by subtracting the axial–equatorial conformational preference of the substituted cyclohexane Ch **III**–**X** from that of the corresponding THP **I**–**X** by using the total molecular electronic energies of both systems. By contrast, $\xi E_{total}^{OCX}$ is a within-THP atomic energy contribution obtained by summing the RIAE terms assigned to the O–C–X fragment for the axial–equatorial comparison of a single THP. Thus, τ estimates the excess axial stabilization of the THP relative to the external cyclohexane reference, whereas $\xi E_{total}^{OCX}$ describes the contribution assigned by the RIAE partition to the O–C–X fragment within the THP itself. Consequently, τ and $\;\xi E_{total}^{OCX}$ are complementary but distinct quantities; they are neither numerically equivalent nor expected to coincide.

As shown in Table 6, the RIAE ξ$E_{total}^{ae}$ total energies reproduce the stability trends obtained from the full M06-2X/6-311G(d,p) gas-phase optimizations (see Table 1). In all six THPs **I**–**X**, the O–C–X framework provides a stabilizing contribution to the axial conformation, whereas the remaining hydrocarbon framework contributes unfavorably and partially offsets this stabilization. Within the halogen series, the axial stabilization becomes progressively more favorable from F to Cl and Br, with values of −3.19, −4.70, and −5.31 kcal·mol^−1^, respectively. The methoxy derivative **I**–**O** shows greater axial stabilization (−4.04 kcal·mol^−1^) than the dimethylamino derivative **I**–**N** (−2.64 kcal·mol^−1^), whereas **I**–**S** displays the weakest axial stabilization of the series (−2.26 kcal·mol^−1^).

Decomposition of the O–C–X contribution further reveals a period-associated change in the dominant electronic term. For compounds containing second-period X elements (X = N, O, and F), the relative intra-atomic energies are strongly stabilizing, ranging from −17.04 to −11.12 kcal·mol^−1^, whereas the corresponding interatomic contributions are destabilizing. In contrast, for compounds containing third- and fourth-period X elements (X = S, Cl, and Br), the interatomic contributions become strongly stabilizing, ranging from −34.05 to −7.72 kcal·mol^−1^. Thus, the RIAE decomposition identifies two distinct energetic regimes: axial stabilization is dominated by intra-atomic terms for second-period X elements and by interatomic terms for third- and fourth-period X elements.

A detailed analysis of IQA $E_{intra}^{A}$ intra-atomic energies of the atoms contributing to the relative ξ$E_{intra}^{OCX}$ intra-atomic energies of **I**–**N**, **I**–**O**, and **I**–**F** (second-period X elements) indicates that the relative $E_{intra}^{C2}$ intra-atomic energies at the anomeric C2 carbon, −9.25, −13.76, and −15.33 kcal·mol^−1^, respectively, are the dominant electronic factors at an atomic level for the axial stabilization of these THPs (see Table 7 and Table S11 of the Supplementary Materials). Note that this stabilization increases with the electronegativity of the X atom. Examination of the IQA components further reveals that the relatively favorable nuclei-electron contributions between the axial and equatorial conformations, −58.56 (**I**–**N**), −79.36 (**I**–**O**), and −70.37 (**I**–**F**) kcal·mol^−1^, constitute the dominant contribution to this intra-atomic anomeric stabilization (see Table 7). A comparable intra-atomic stabilization at the anomeric carbon was recently reported by Popelier for the fluorine derivative **I**–**F** using IQA and REG analyses [21].

Conversely, for compounds containing third- and fourth-period X elements (X = S, Cl, and Br), analysis of the IQA $E_{inter}^{AB}$ interatomic energies of the atoms contributing to ξ$E_{inter}^{OCX}$ interatomic energies of **I**–**S**–**a**, **I**–**Cl**–**a**, and **I**–**Br**–**a** indicates that the $E_{inter}^{AB}$ interatomic energies associated with O1 and C2 atoms are stabilizing. The corresponding O1 and C2 contributions, −2.47 and −2.36 (**I**–**S**–**a**), −16.06 and −8.89 (**I**–**Cl**–**a**), and −20.94 and −12.14 (**I**–**Br**–**a**) kcal·mol^−1^, drive axial stabilization (see Table S11 of the Supplementary Materials). A further analysis of the IQA $E_{inter}^{AB}$ terms shows that the $E_{inter}^{AB}$ energies between O1 and C2, −12.45 (**I**–**S**–**a**), −36.23 (**I**–**Cl**–**a**), and −48.90 kcal·mol^−1^ (**I**–**Br**–**a**), constitute the most favorable axial electronic interactions in these sulfur and halogenated systems. This O1 and C2 interatomic stabilization agrees with the reduction of the O1–C2 distance in the more favorable **I**–**X**–**a** compounds containing third- and fourth-period X elements.

The comparatively small ξ$E_{intra}^{A}$ and ξ$E_{inter}^{AB}$ energy differences between the axial and equatorial conformations of **I**–**S** (Table 6) may account for its pronounced deviation in Figure 3, suggesting that additional intramolecular interactions contribute with magnitudes comparable to that associated with the *anomeric effect*.

**Figure 8:**
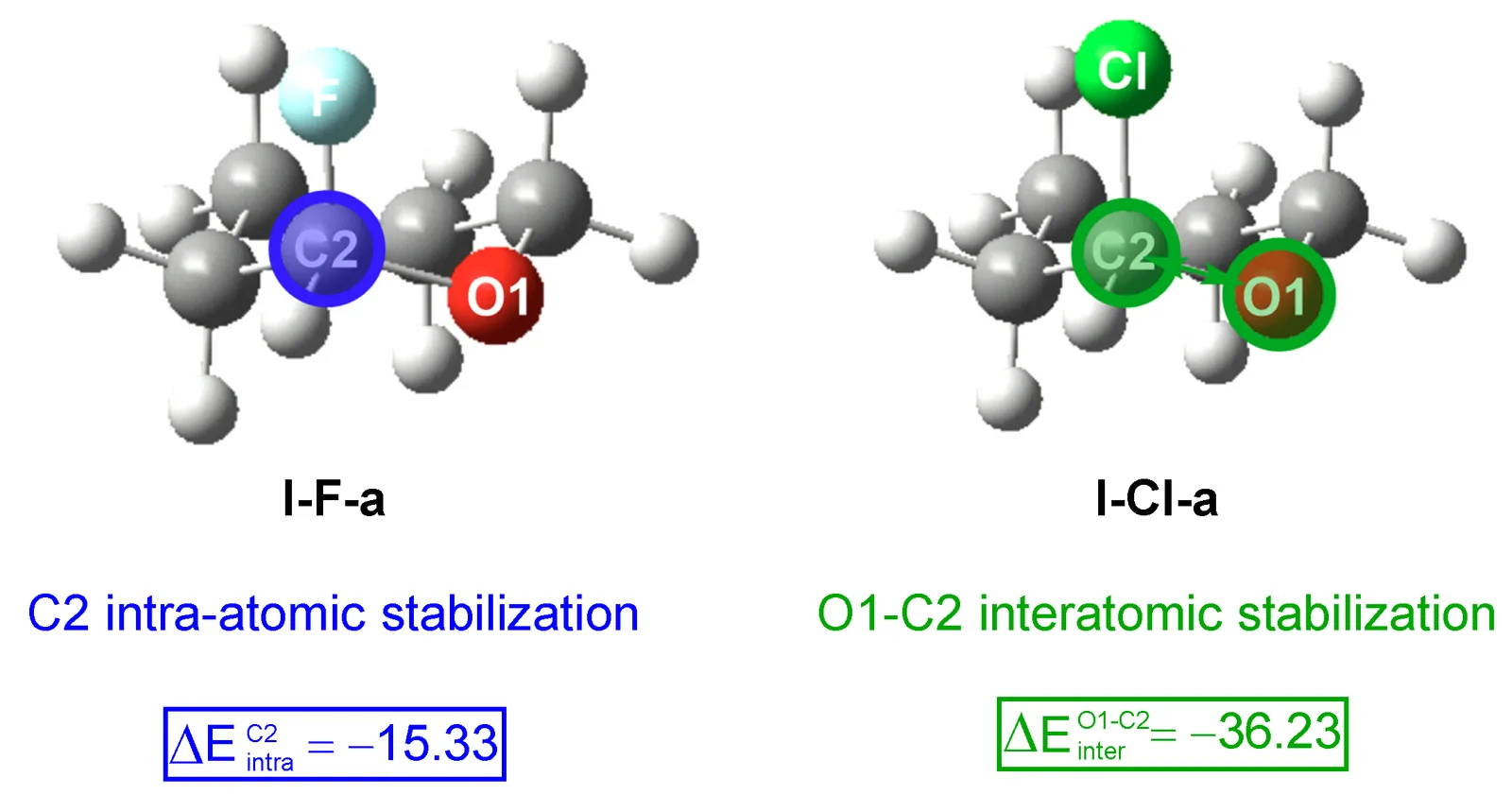
Graphical representation of the C2 intra-atomic stabilization (in blue) for THPs containing second-period X elements, and the O1–C2 interatomic stabilization (in green) for THPs containing third- and fourth-period X elements. Relative IQA axial-equatorial energies ΔE are given in kcal·mol^−1^.

The RIAE analysis reveals a period-associated change in the distribution of the energy contributions responsible for the axial stabilization of THPs **I**–**X**. On this basis, the studied compounds can be classified into two groups: (i) Group I comprises THPs containing second-period X elements (X = N, O, and F), for which stabilization of the O–C–X framework is dominated by favorable intra-atomic contributions assigned to the anomeric C2 atom; and (ii) Group II comprises THPs containing third- and fourth-period X elements (X = S, Cl, and Br), for which the dominant stabilizing contribution is the interatomic interactions between the ether O1 oxygen and the anomeric C2 carbon. Thus, the RIAE decomposition identifies a shift in the dominant energy contribution from an intra-atomic C2 term in Group I to an interatomic O1–C2 term in Group II. Figure 8 summarizes the C2 intra-atomic and O1–C2 interatomic electronic stabilizations for the axial preference of THPs containing second-period X elements (Group I), and for compounds containing third- and fourth-period X elements (Group II), respectively, which are responsible at the atomic level for the *anomeric effect* characterized in this RIAE study.

A comparison of the RIAE decomposition for the cyclic THPs **I**–**X** (Table 6) with that obtained for the acyclic MMs **II**–**X** (Table S4 of the Supplementary Materials) reveals the same qualitative period-associated pattern. Only MMs **II-N** shows an exception as the RIAE $E_{total}^{ae}$ total energies, −3.20 kcal·mol^−1^, arises from low stabilizing ξ$E_{intra}^{A}$ intra-atomic and ξ$E_{inter}^{AB}$ interatomic energies, −1.75 and −1.45 kcal·mol^−1^, respectively.

Analysis of the relative ξ$E_{intra}^{A}$ intra-atomic and ξ$E_{inter}^{AB}$ interatomic energies contributing to the RIAE ξ$E_{total}^{ae}$ total energies for the series of acyclic MMs **II**–**X** shows that, except for MMs **II-N**, the other five MMs present the same dependence on the period of the X atom as that observed for the cyclic THPs **I-X** series.

This agreement shows that the two patterns of RIAE contributions are reproduced in both cyclic and acyclic O–C–X frameworks. The smaller axial stabilization obtained for THPs **I**–**X** relative to the 60-DA stabilization of MMs **II**–**X** is associated with the unfavorable relative total-energy contribution assigned to the hydrocarbon CH framework of the cyclic systems, ξ$E_{total}^{CH}$, which ranges from 1.57 to 5.39 kcal·mol^−1^ (Table 6). This positive contribution partially offsets the stabilization associated with the O–C–X framework in the THP series.

Finally, analysis of the relative axial/equatorial IQA $E_{intra}^{A}$ intra-atomic and $E_{inter}^{AB}$ interatomic energies associated with the X element in the series of THPs **I**–**X** were analyzed (see Table S11 of the Supplementary Materials). Except in the cases of **I**–**N** and **I**–**O**, where the relative $E_{inter}^{AB}$ interatomic energies are stabilizing, the remaining systems show an axial intra-atomic stabilization at X, ranging from −3.05 (**I**–**S**) to −10.17 (**I**–**Br**) kcal·mol^−1^. Note that, in the case of the Br atom, which experiences high $E_{intra}^{A}$ intra-atomic stabilization, this represents only ca. 25% of the $E_{inter}^{AB}$ interatomic stabilization associated with the O1 and C2 atoms. These $E_{intra}^{A}$ intra-atomic stabilizations cannot be correlated with the atomic charges seen at the X atom, which differ between compounds containing second-period and third- or fourth-period X elements (see Table 5). Note that at the THPs **I**–**F** and **I**–**Cl,** the halogen X atoms experience an axial $E_{intra}^{A}$ intra-atomic stabilization of ca. 5 kcal·mol^−1^ despite having markedly different atomic charges of −0.42 and −0.16 e, respectively (see Table 5). Consequently, while the stabilizations of the O1 and C2 atoms at an atomic level may be related to the X element period, those associated with the X atom, which have a minor contribution to the *anomeric effect*, show less systematic behavior and have lower weight in the axial stabilization.

**Figure 9:**
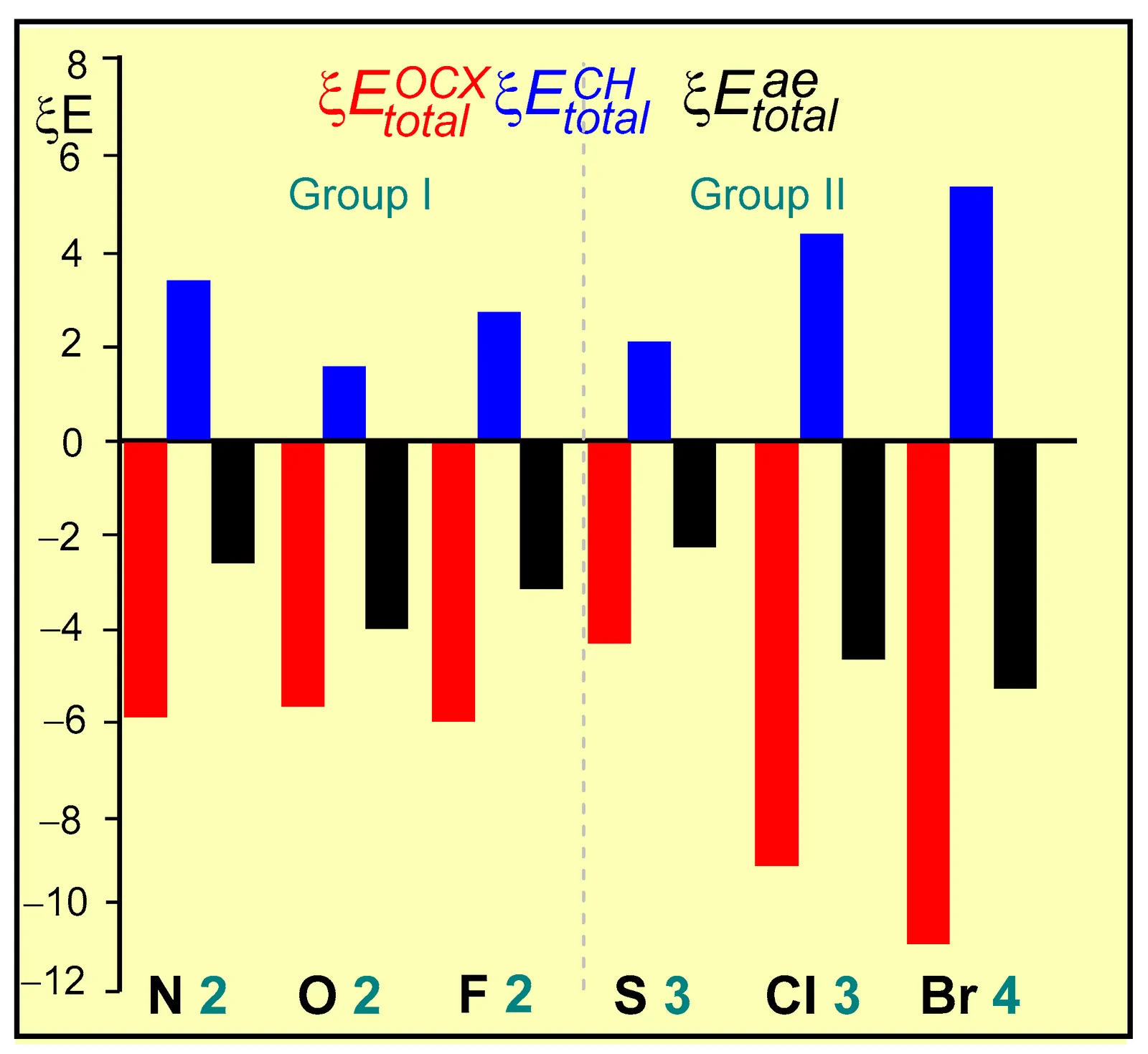
Graphical representation of the sets of the M06-2X/6-311G(d,p) gas-phase relative ξ$E_{total}^{OCX}$, ξ$E_{total}^{CH}$, and ξ$E_{total}^{ae}$ total energies between the axial and equatorial conformations of 2-substituted THPs **I**–**X**. The relative ξ$E_{total}^{ae}$ energies correspond to the RIAE energies between the two stereoisomers. Relative ξ$E_{total}^{X}$ energies for the OCX and CH frameworks are shown in red and blue, respectively, while the black bar represents the relative ξ$E_{total}^{ae}$ energies. All ξ$E_{total}^{X}$ energies are reported in kcal mol^−1^. The period position of the X atom is shown in green.

Figure 9 provides a graphical summary of the RIAE decomposition reported in Table 6. It highlights the stabilizing contribution of the O–C–X framework to the axial conformations and the opposing destabilizing contribution of the remaining hydrocarbon framework. The figure also illustrates the progressive increase in axial stabilization along the halogen series F < Cl < Br. The detailed atomic origin of these trends is discussed above.

#### 2.5.2. RIAE Analysis of Methoxycyclohexane **III**–**O** and Methylcyclohexane **III**–**Me**

Methoxycyclohexane Ch **III**–**O**, which lacks the O–C–X motif, displays a significant axial stabilization of −2.56 kcal·mol^−1^, in contrast to the expected axial destabilization of methylcyclohexane **III**–**Me**, 1.50 kcal·mol^−1^ (Table 1). This intrinsic axial preference associated with the methoxy group also contributes in concert with the *anomeric effect*, stabilizing the axial THP **I**–**O** and enhancing its overall axial stabilization to 4.38 kcal·mol^−1^. The corresponding τ value for oxygen is 1.82 kcal·mol^−1^ (Table 1 and Figure 4), indicating that both intrinsic substituent effects and the anomeric contribution participate in the stabilization of THP **I**–**O**.

To identify the electronic interactions underlying the axial stabilization of methoxycyclohexane **III**–**O**, a RIAE analysis was performed for Ch **III**–**O** and methylcyclohexane **III**–**Me** as reference systems. The M06-2X/6-311G(d,p) gas-phase relative intra-atomic, ξ$E_{intra}^{A}$ intra-atomic, ξ$E_{inter}^{AB}$ interatomic, and ξ$E_{total}^{ae}$ total energy differences between the axial and equatorial conformations of Chs **III**–**O** and **III**–**Me** are given in Table 8.

Analysis of the relative ξ$E_{intra}^{A}$ intra-atomic and ξ$E_{inter}^{AB}$ interatomic energies responsible for the axial and equatorial stabilizations of Chs **III**–**O** and **III**–**Me** indicate that in both Chs, the ξ$E_{intra}^{A}$ intra-atomic energies are destabilizing, while the ξ$E_{inter}^{AB}$ interatomic energies are stabilizing. However, in Ch **III**–**O,** the relative ξ$E_{inter}^{AB}$ interatomic energies are stabilizing at −3.75 kcal·mol^−1^, yielding an axial stabilization of −2.65 kcal·mol^−1^; in Ch **III**–**Me**, the relative ξ$E_{intra}^{A}$ intra-atomic energies are more destabilizing at 4.13 kcal·mol^−1^, yielding an axial destabilization of 1.46 kcal·mol^−1^ (see Table 8). A detailed analysis of the relative $E_{inter}^{AB}$ interatomic energies of the atoms contributing to ξ$E_{inter}^{AB}$ interatomic energies of Ch **III**–**O**–**a** indicates that the $E_{inter}^{AB}$ interatomic energies associated with the ether O oxygen, −8.88 kcal·mol^−1^, drive axial stabilization.

Interestingly, the dominant energetic factors underlying axial stabilization in Ch **III**–**O** and THP **I**–**O** differ qualitatively. In Ch **III**–**O**, axial stabilization is dominated by favorable interatomic interactions associated with the ether O oxygen, whereas in THP **I-O**, this arises primarily from favorable intra-atomic contributions assigned to the anomeric C2 atom, consistent with the RIAE pattern identified for Group I compounds containing second-period X elements. This contrast shows that different distributions of atomic energy contributions can lead to axial stabilization in related systems and helps distinguish the contribution associated with the O–C–X motif from the other electronic contributions involved in overall stabilization.

## 3. Conclusions

The *anomeric effects* associated with the O–C–X motif in 2-substituted cyclic and acyclic ethers have been analyzed within the MEDT framework by using recently introduced RIAE analysis at the M06-2X/6-311G(d,p) level. This approach has been applied to two representative series of 2-substituted ethers: six cyclic THPs **I**–**X** and six acyclic MMs **II**–**X** (X = N, O, F, S, Cl, and Br).

The computed conformational energies favor the axial conformers of THPs **I**–**X** and the 60-DA conformers of MMs **II**–**X**. However, the total axial stabilization of the THPs should not be equated with the contribution attributed to the O–C–X anomeric motif. A comparison with the corresponding substituted Chs defines the anomeric τ index as the excess axial stabilization of the THP relative to its cyclohexane reference. This descriptor, which is found to depend on the computational level, provides an operational estimate of the contribution associated with the O–C–X motif, assuming an approximate cancelation of common substituent–framework effects. It is distinct from the atomic energy contribution assigned to the OCX fragment by the RIAE partition of the THP itself. The resulting τ values reveal a systematic dependence on the identity of X and allow their variations to be examined in relation to the group and period of the X element.

Although geometrical descriptors, NPA charges, and ELF analyses reveal systematic signatures associated with axial conformational stabilization, these structural and electron-density features alone do not establish their energetic origins. The RIAE analysis provides a complementary energetic description and identifies two distinct patterns in the distribution of stabilizing atomic energy contributions that correlate with the period of X in the systems examined. For second-period substituents (X = N, O, and F; Group I), stabilization is dominated by favorable intra-atomic contributions centered at the anomeric C2 carbon. For third- and fourth-period substituents (X = S, Cl, and Br; Group II), the dominant stabilization arises from favorable interatomic contributions between the ether O1 and anomeric C2 atoms. The energetic contribution associated directly with the X atom is comparatively smaller and less systematic. Analogous RIAE patterns are observed in the cyclic and acyclic series.

RIAE analysis of Ch **III**–**O** further illustrates that axial stabilization can be associated with different distributions of atomic energy contributions. In Ch **III**–**O**, the dominant stabilizing contributions are interatomic terms involving methoxy oxygen, while in THP **I**–**O**, stabilization arises primarily from favorable intra-atomic contributions at the anomeric C2 carbon. This contrast reinforces the distinction between the overall conformational preference and the individual atomic energy contributions underlying it.

Consequently, this MEDT/RIAE study provides a quantitative atomic energy framework for understanding the *anomeric effect*, combining the reference-based τ descriptor for the THP series with complementary RIAE analyses of the cyclic and acyclic systems examined.

## 4. Computational Details

The M06-2X density functional [35], together with the 6-311G(d,p) basis set [36], was used for the geometry optimizations, conformational-energy calculations, solvent calculations, and ELF [31] and IQA [19] analyses reported in this study. The 6-311G(d,p) basis set is a triple-split-valence basis augmented with polarization functions on both non-hydrogen and hydrogen atoms. To assess the sensitivity of the gas-phase axial–equatorial conformational energies to the selected density functional and the use of diffuse functions, additional geometry optimizations and conformational-energy calculations were performed using B3LYP [37,38] functional with the 6-311G(d,p) basis set. The B3LYP/6-311G(d,p) and M06-2X/6-311++G(d,p) levels were used only for a methodological comparison, and were not employed in the RIAE decomposition. All electronic structure calculations were carried out using the Gaussian 16 suite of programs [39].

Solvent effects were incorporated by fully reoptimizing the gas-phase stationary points at the same theoretical level by using the polarizable continuum model (PCM) [40,41] within the self-consistent reaction field (SCRF) framework [42,43,44]. ELF analyses [31] of the M06-2X/6-311G(d,p) monodeterminantal wavefunctions were performed with the TopMod [45] package by employing a cubic grid with a step size of 0.1 Bohr. Molecular geometries and ELF basin attractors were visualized using the GaussView program [46]. IQA analysis was performed using the AIMAll package [47] and the corresponding M06-2X/6-311G(d,p) monodeterminantal pseudo-wavefunctions. Within this framework, the total electronic energy is partitioned into intra-atomic and interatomic contributions. For the DFT calculations, the exchange-correlation energy (VXC) components are evaluated through unambiguous numerical integration over the respective Bader atomic basins (Ω), as implemented by Keith [47]. The scope of these components is inherently bound to this specific topological partitioning scheme. The boundaries of the interatomic surfaces (IAS) were resolved using the superfine mesh algorithm (-iasmesh=superfine), which restricts the maximum deviation between adjacent vector trajectories to less than 10% of the target spacing.

The axial and equatorial conformations of F−, Cl−, and Br–substituted THPs and Chs were fully optimized in their most stable chair forms. For the NMe_2_−, OMe−, and SMe–substituted derivatives, the conformers generated by rotation around the exocyclic C2–X bond were thoroughly examined, and the lowest-energy axial and equatorial conformers were selected for subsequent analyses. The Cartesian coordinates of all optimized geometries are provided in the Supplementary Material.

## Figures and Tables

**Table 1:** M06-2X/6-311G(d,p) gas-phase relative energies, ΔE_e−a_ in kcal·mol^−1^, between the axial and equatorial conformations of cyclic 2-substituted THPs **I**–**X**, Δ$E_{e-a}^{THP}$, the 60- and 180-DA alternate conformations of acyclic substituted MMs **II**–**X**, Δ$E_{e-a}^{MM}$, and the axial and equatorial conformations of substituted Chs **III**–**X**, Δ$E_{e-a}^{Ch}$. The anomeric τ index, in kcal·mol^−1^, quantifies the contribution of the *anomeric effect* to the axial stabilization of 2-substituted THPs **I**–**X**.

THP–X	Δ$E_{e-a}^{THP}$	MM–X	Δ$E_{e-a}^{MM}$	Ch–X	Δ$E_{e-a}^{Ch}$	τ
**I**–**N**	2.80	**II**–**N**	3.14	**III**–**N**	1.06	1.74
**I**–**O**	4.38	**II**–**O**	3.19	**III**–**O**	2.56	1.82
**I**–**F**	3.23	**II**–**F**	4.59	**III**–**F**	0.42	2.81
**I**–**S**	2.28	**II**–**S**	2.79	**III**–**S**	−0.24	2.52
**I**–**Cl**	4.26	**II**–**Cl**	5.28	**III**–**Cl**	−0.07	4.33
**I**–**Br**	4.94	**II**–**Br**	5.74	**III**–**Br**	−0.01	4.95
**I**–**Me**	−2.42			**III**–**Me**	−1.50	
				**III**–**tBu**	−4.55	

**Table 2:** Dielectric constants ε of the solvents, relative energies of the axial and equatorial conformations with respect to the gas phase Δ$E_{ax}^{s}$ and Δ$E_{eq}^{s}$, and relative axial-equatorial energies in the different solvents, Δ$E_{ae}^{s\;}$. M06-2X/6-311G(d,p) relative energies are given in kcal·mol^−1^.

Solvent	Dielectric Constant ε	Δ$E_{ax}^{s}$	Δ$E_{eq}^{s}$	Δ$E_{ae}^{s}$ ^(a)^
vacuo	0.0	0.00	0.00	−4.38
benzene	2.3	−1.15	−1.53	−4.01
chloroform	4.7	−1.85	−2.48	−3.76
acetone	20.7	−2.50	−3.39	−3.50
acetonitrile	37.5	−2.60	−3.53	−3.46
water	78.5	−2.67	−3.63	−3.43

^(a)^ ${∆E}_{ae}^{s\;}$ values correspond to solvent-phase axial-equatorial energy differences, defined by Δ$E_{ae}^{s}=\;∆E_{ae}^{gas}+(\Delta E_{ax}^{s}-\Delta E_{eq}^{s})$.

**Table 3:** M06-2X/6-311G(d,p) relative axial/equatorial electronic energies, enthalpies, and Gibbs free energies Δ$E_{ae}^{s}$, Δ$H_{ae}^{s}$, and Δ$G_{ae}^{s}$ in kcal·mol^−1^, and entropies Δ$S_{ae}^{s}$ in cal·mol^−1^·K^−1^, computed in solvent at room temperature, for the axial/equatorial equilibrium of 2-methoxy THP **I**–**O**.

Solvent	Δ$E_{ae}^{s}$	Δ$H_{ae}^{s}$	Δ$S_{ae}^{s}$	Δ$G_{ae}^{s}$	Δ(Δ$E_{ae}^{s}$ − Δ$G_{ae}^{s}$)
benzene	−4.01	−3.92	0.28	−4.00	−0.01
acetone	−3.50	−3.43	0.03	−3.44	−0.06
water	−3.43	−3.36	0.00	−3.36	−0.07

**Table 4:** O1–C2 and C2–X bond-length differences, Δl_a−e_ = l_axial_ − l_equatorial_, for the 2-substituted THPs **I**–**X**. Bond-length differences are given in Å.

	O1–C2	C2–X
**I**–**N**	−0.001	0.015
**I**–**O**	−0.006	0.015
**I**–**F**	−0.013	0.028
**I**–**S**	−0.007	0.028
**I**–**Cl**	−0.023	0.060
**I**–**Br**	−0.029	0.080

**Table 5:** M06-2X/6-311G(d,p) natural atomic charges at the O1, C2, and X atoms of the axial and equatorial conformations of 2-substituted THPs **I**–**X**. Atomic charges are given in units of the elementary charge (e).

	**I**–**N**–**a**	**I**–**O**–**a**	**I**–**F**–**a**	**I**–**S**–**a**	**I**–**Cl**–**a**	**I**–**Br**–**a**
O1	−0.61	−0.62	−0.60	−0.60	−0.58	−0.57
C2	0.34	0.46	0.56	0.04	0.21	0.17
X	−0.56	−0.61	−0.42	0.12	−0.16	−0.14
	**I**–**N**–**e**	**I**–**O**–**e**	**I**–**F**–**e**	**I**–**S**–**e**	**I**–**Cl**–**e**	**I**–**Br**–**e**
O1	−0.61	−0.62	−0.60	−0.61	−0.60	−0.60
C2	0.34	0.48	0.58	0.02	0.17	0.12
X	−0.53	−0.58	−0.39	0.18	−0.07	−0.01

**Table 6:** M06-2X/6-311G(d,p) gas-phase axial/equatorial relative intra-atomic, interatomic, and total RIAE contributions ξ$E_{intra}^{X}$, ξ$E_{inter}^{X}$, and ξ$E_{total}^{X}$, respectively, for the OCX and CH frameworks of 2-substituted THPs **I**–**X**. Energies are given in kcal·mol^−1^. The ξ$E_{total}^{OCX}$ total energies provide axial stabilization associated with the energetic component of the *anomeric effect*, while the sum of the ξ$E_{total}^{X}$ energies of both frameworks, i.e., ξ$E_{total}^{ae}$ gives the total RIAE axial stabilization.

I–X Period	ƒ(X)	ξ$E_{intra}^{X}$	ξ$E_{inter}^{X}$	ξ$E_{total}^{X}$	ξ$E_{total}^{ae}$
**I**–**N** 2	OCX	−11.14	5.21	−5.92	−2.64
	CH	−4.75	8.03	3.28	
**I**–**O** 2	OCX	−11.12	5.52	−5.61	−4.04
	CH	−3.99	5.56	1.57	
**I**–**F** 2	OCX	−17.04	11.09	−5.95	−3.19
	CH	−1.64	4.40	2.76	
**I**–**S** 3	OCX	3.42	−7.72	−4.31	−2.26
	CH	−1.38	3.43	2.05	
**I**–**Cl** 3	OCX	18.33	−27.37	−9.04	−4.70
	CH	1.01	3.34	4.34	
**I**–**Br** 4	OCX	23.35	−34.05	−10.70	−5.31
	CH	1.25	4.14	5.39	

**Table 7:** M06-2X/6-311G(d,p) gas-phase IQA $E_{intra}^{A}$ intra-atomic energies, in a.u., and the relative Δ$E_{intra}^{C2}$ intra-atomic energies, in kcal·mol^−1^, of C2 carbon of the axial and equatorial conformations of the nitrogen, fluorine, and oxygen 2-substituted THPs **I**–**X**.

	$IQA\;E_{intra}^{A}$	T(A)	Vne(A)	Vee(A)
**I**–**N**–**a**	−37.2795	37.4094	−86.7139	12.0249
**I**–**N**–**e**	−37.2648	37.3969	−86.6205	11.9589
Δ$E_{intra}^{C2}$	−9.25	7.86	−58.56	41.45
**I**–**O**–**a**	−37.1487	37.2819	−85.8739	11.4433
**I**–**O**–**e**	−37.1268	37.2585	−85.7474	11.3622
Δ$E_{intra}^{C2}$	−13.76	14.70	−79.36	50.90
**I**–**F**–**a**	−37.0988	37.2438	−85.6188	11.2762
**I**–**F**–**e**	−37.0744	37.2244	−85.5067	11.2079
Δ$E_{intra}^{C2}$	−15.33	12.17	−70.37	42.87

**Table 8:** M06-2X/6-311G(d,p) gas-phase relative ξ$E_{intra}^{A}$ intra-atomic, ξ$E_{inter}^{AB}$ interatomic, and ξ$E_{total}^{ae}$ total energies (in kcal·mol^−1^) between the axial and equatorial conformations of Chs **III**–**O** and **III**–**Me**.

Ch	ξ$E_{intra}^{A}$	ξ$E_{inter}^{AB}$	ξ$E_{total}^{ae}$
**III**–**O**	1.10	−3.75	−2.65
**III**–**Me**	4.13	−2.67	1.46

## Data Availability

The original contributions presented in this study are included in the article/Supplementary Material. Further inquiries can be directed to the corresponding authors.

## Supplementary Materials

The following supporting information can be downloaded at: https://www.mdpi.com/article/10.3390/molecules31183335/s1.
