# Supplementary material for: Relative Interacting Atomic Energy Analysis of the Anomeric Effect in Cyclic and Acyclic O–C–X Systems
Source: Molecules. 2026 Sep 20;31(18):3335. doi: 10.3390/molecules31183335 (PMC13610088; doi:10.3390/molecules31183335)
Supplement: Supplementary file 1 [file molecules-31-03335-s001.zip › molecules-4545439-supplementary.pdf]

Supplementary Material

Relative Interacting Atomic Energy Analysis of the Anomeric Effect in Cyclic and Acyclic O–C–X Systems

Luis R. Domingo,[1]* Mar Ríos-Gutiérrez,[2] and Patricia Pérez[3]*

[1] Independent Researcher, Avd. Tirso de Molina 20, 46015, Valencia, Spain.
[2] Department of Organic Chemistry, University of Valencia, Dr. Moliner 50, 46100 Burjassot, Spain.
[3] Facultad de Ciencias, Escuela de Química y Farmacia, Universidad San Sebastián, Campus Ciudad Universitaria, Av. del Cóndor 720, Ciudad Empresarial, Huechuraba, Santiago, 8580704, Chile.

* Correspondence: luisrdomingo@gmail.com (L.R.D.)
patricia.perez@uss.cl (P.P.)

Index

Section S1. Dissecting the IQA E_{inter}^{AB} interatomic energies

Section S2. Energy analysis of the 60-/180-DA conformations of acyclic substituted MMs **II-X**.

Section S3. Geometrical and atomic charge analyses of acyclic substituted MMs **II-X**.

Section S4. RIAE analysis of the acyclic substituted MMs **II-X**.

Table with the M06-2X/6-311G(d,p) gas-phase total energies of the axial and equatorial conformations of cyclic 2-substituted THPs **I-X**, and the 60- and 180-DA conformations of acyclic substituted MMs **II-X**.

Table with the M06-2X/6-311G(d,p) gas-phase total energies of the axial and equatorial conformations of substituted Chs **III-X**.

Table with the M06-2X/6-311G(d,p) gas-phase relative axial/equatorial E_{intra}^{A} intra-atomic and E_{inter}^{AB} interatomic energies associated with the O1, C2 and X atoms of THPs **I-X**.

M06-2X/6-311G(d,p) computed total energies and Cartesian coordinates of the axial and equatorial conformations of cyclic 2-substituted THPs **I-X**.

M06-2X/6-311G(d,p) computed total energies and Cartesian coordinates of the 60- and 180-DA conformations of acyclic substituted MMs **II-X.**

M06-2X/6-311G(d,p) computed total energies and Cartesian coordinates of the axial and equatorial conformations of substituted CHs **III-X**.

Section S1. *Dissecting the IQA E_{inter}^{AB} interatomic energies.*

Many organic molecules, such as cyclic hydrocarbons, tetrahydropyrans (THPs), and pyranoses, exist in some stereoisomeric forms that differ in their three-dimensional molecular geometries and total electronic energies. According to Interacting Quantum Atoms (IQA), the Kohn-Sham (KS) energies can be partitioned into two main contributions: (i) the E_{intra}^{A} intra-atomic energies, associated with the kinetic and electrostatic energies of the A atom; and (ii) the E_{inter}^{AB} interatomic energies, associated with the electrostatic electronic interactions between the A atom and the B atom. In turn, the E_{inter}^{AB} interatomic energies can be further decomposed into: (ii-a) primary interatomic energies, associated with bonding interactions between two adjacent atoms (A,B); and (ii-b) secondary interatomic energies, corresponding to interactions between non-bonded atoms (A,C). The latter can be attractive or repulsive, and they are generally considered responsible for the energy differences between stereoisomers. Therefore, the axial/equatorial energy difference in THPs and pyranoses (Figure 1) arises not only from the classical *anomeric effect* associated with the O–C–X motif, but also from additional secondary interatomic energies present in these systems.

Table S1 reports an IQA energy decomposition analysis of the KS energies of fluoro(methoxy)methane **I-F-a**. Some interesting conclusions can be drawn from this analysis: (i) a deviation of 9.1 10^{-5} a.u. is found between the M06-2X/6-311G(d,p) total KS and the total IQA energies, confirming the internal consistency of the IQA partitioning; (ii) the total IQA E_{intra}^{A} intra-atomic energies, which represent the sum of the intra-atomic energies of the seven atoms that constitute **I-F-a,** account for 98.79% of the total KS energy. This means that practically all molecular energy is associated with the specific atomic energies; (iii) the total IQA E_{inter}^{AB} interatomic energies represent only the 1.21% of the total KS energy; (iv) the total IQA E_{inter}^{AB} interatomic energies between the adjacent (A,B) atoms, −3.178184 a.u., which include the forces that hold the atoms together in the molecule, represent only 1.25 % of the total KS energy; and finally (v) the total IQA E_{inter}^{AC} interatomic energies between non-adjacent (A,C) atoms, 0.096112 a.u., which include the electronic interactions between non-bonded atoms within **I-F-a**, play a key role in determining conformational preferences. The E_{inter}^{AC} interatomic energies represent 0.04% of the total KS energy, and they are positive and, consequently, unfavorable (see Table S1). These results highlight that subtle non-bonded interatomic

interactions although energetically small, are decisive in controlling stereochemical preferences in systems exhibiting the *anomeric effect*.

Table S1. M06-2X/6-311G(d,p) total KS energies of fluoro(methoxy)methane **I-F-a**, total IQA energies, total IQA E_{intra}^{A} intra-atomic energies; total IQA E_{inter}^{AB} interatomic energies; total IQA E_{inter}^{AB} interatomic energies between continuous AB atoms, and total E_{inter}^{AC} IQA interatomic energies between non-continuous AC atoms. Energies are given in a.u..

	E
KS energies	-254.238663
IQA energies	-254.238572
IQA E_{intra}^{A} energies	-251.156401
IQA E_{inter}^{AB} energies	-3.082171
IQA E_{inter}^{AB} energies (AB)	-3.178184
IQA E_{inter}^{AC} energies (AC)	0.096112

Section S2. *Energy analysis of the 60-/180-DA conformations of acyclic substituted MMs **II-X**.*

The 60-/180-degree alternate (DA) conformers of the six methoxymethanes (MMs) **II–X**, **X = N, O, F, S, Cl, Br**, were optimized at the M06-2X/6-311G(d,p) level in the gas phase. The resulting ΔE^{MM}_{e-a} relative energies are presented in Table S2. The optimized geometries are given in Figure S1. The total energy is given in Table S7.

For the acyclic MMs **II–X**, the 60-DA conformers are favored over the 180-DA ones by 2.79 (**II-S**) – 5.74 (**II-Br**) kcal·mol^{-1}. For the halogen series, **X** = F, Cl, and Br, the 60-DA stabilization follows the trend F < Cl < Br, indicating that *anomeric effect* increases as the period of the X halogen increases. In contrast, within the N, O, and F series, as well as within the S and Cl series, the 60-DA stabilization increases with increasing electronegativity of the X atom.

Table S2. M06-2X/6-311G(d,p) gas-phase relative energies, ΔE^{MM}_{e-a} in kcal·mol^{-1}, between the corresponding 180- and 60-DA conformations of acyclic substituted MMs **II-X**.

MM-X	ΔE^{MM}_{e-a}
**II-N**	3.14
**II-O**	3.19
**II-F**	4.59
**II-S**	2.79
**II-Cl**	5.28
**II-Br**	5.74

Section S3. *Geometrical and atomic charge analyses of acyclic substituted MMs **II-X***

The optimized geometries of substituted MMs **II-X** are given in Figure S1. The differences in the O1–C2 and C2–X distances, $\Delta l_{a-e} = l_{axial} - l_{equatorial}$, at the 60- and 180-DA conformations of substituted MMs **II-X** are summarized in Tables S3, while selected natural atomic charges at the O1–C2–X frameworks for acyclic MMs **II–X** are summarized in Table S4.

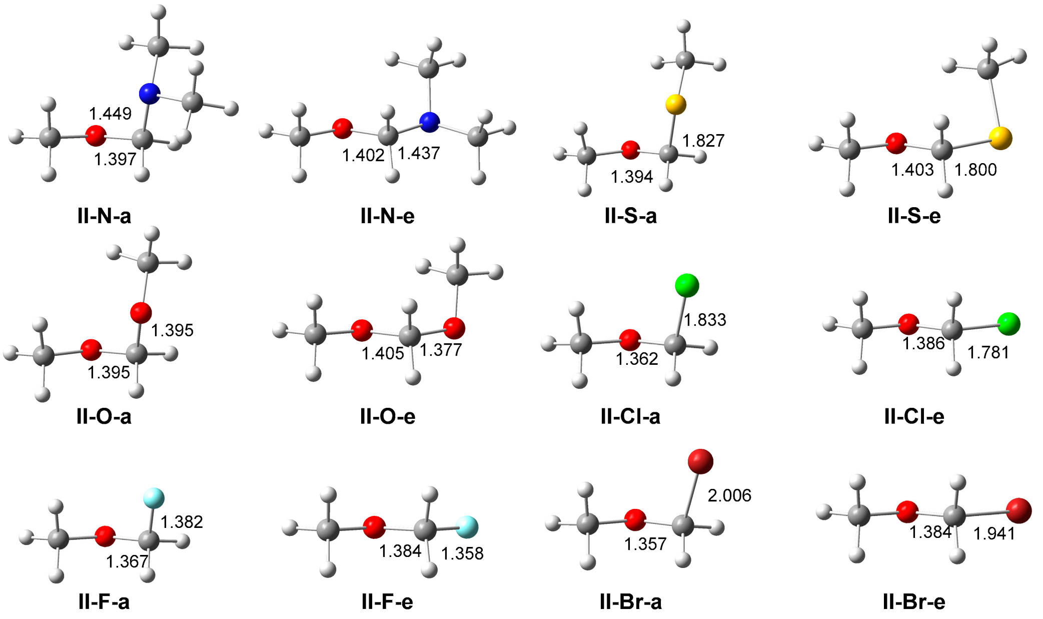

Figure S1. M06-2X/6-311G(d,p) gas-phase optimized geometries of the 60- and 180-DA conformations of substituted MMs **II-X**. The bond lengths are given in angstroms Å.

From the differences in the O1–C2 and C2–X distances given in Table S3, several conclusions arise: (i) while the O1–C2 distances at the axial conformation are reduced between 0.005 and 0.027 Å, the C2–X distances are increased between 0.012 and 0.065 Å. Consequently, C2–X single bonds are more susceptible to the *anomeric effect*; (ii) ~~in~~ this series, the difference of C2–X single bond lengths are larger for X atoms belonging to periods higher than 2 (S, Cl, Br) than for second-period element (N, O, F); and finally, (iii) the differences, Δl_{a-e}, in the O1–C2 and C2–X bond lengths vary systematically as the X atom moves to the left within the same period and upward within a group.

Table S3. Differences in the O1–C2 and C2–X distances, Δl_{a-e} in angstroms Å, at the 60- and 180-DA conformations of substituted MMs **II-X**.

	O1–C2	C2–X
**II-N**	−0.005	0.012
**II-O**	−0.010	0.018
**II-F**	−0.017	0.027
**II-S**	−0.009	0.024
**II-Cl**	−0.024	0.052
**II-Br**	−0.027	0.065

The natural atomic charges at the O1–C2–X framework of the 60- and 180-DA conformations of substituted MMs **II–X** (see Table S4) follow the same trend observed for THPs **I–X** in Table 4, confirming the key influence of the electronic nature of the X atom, particularly its period, on the electron density in the C2–X region and on the axial preference of X atom. In MMs **II–X** series, the C2 carbon is ca. 0.20 e less positive than in THP **I–X** series due to the extra hydrogen atoms on the C2 carbons.

Table S4. M06-2X/6-311G(d,p) natural atomic charges at the C3–O1–C2–X atoms at the 60- and 180-DA conformations of substituted MMs **II-X**. The natural atomic charges are reported in average number of electrons, e.

	**II-N-a**	**II-O-a**	**II-F-a**	**II-S-a**	**II-Cl-a**	**II-Br-a**
O1	-0.58	-0.59	-0.57	-0.57	-0.55	-0.54
C2	0.17	0.30	0.41	-0.14	0.02	-0.02
X	-0.54	-0.59	-0.41	0.13	-0.14	-0.12
	II-N-e	II-O-e	II-F-e	II-S-e	II-Cl-e	II-Br-e
O1	-0.58	-0.59	-0.57	-0.58	-0.57	-0.57
C2	0.17	0.31	0.42	-0.16	0.00	-0.06
X	-0.52	-0.57	-0.37	0.18	-0.06	-0.01

Section S4. *RIAE analysis of the acyclic substituted MMs **II-X***

The Relative Interacting Atomic Energy (RIAE) analysis of the six acyclic substituted MMs **II–X** complements the trends observed for the cyclic THPs, allowing to assess whether the electronic origin of the *anomeric effect* persists in the acyclic O–C–X framework. Table S5 summarizes the relative ξE_{intra}^{A} intra-atomic, ξE_{inter}^{AB} interatomic, and ξE_{total}^{ae} total energy differences between the 60- and 180-DA conformations of MMs **II–X**.

Table S5. M06-2X/6-311G(d,p) gas-phase relative ξE_{intra}^{A} intra-atomic, ξE_{inter}^{AB} interatomic and ξE_{total}^{ae} total energies, in kcal·mol^{-1}, between the 60- and 180-DA conformations of substituted MMs **II-X**.

**II-X**	period	ξE_{intra}^{A}	ξE_{inter}^{AB}	ξE_{total}^{ae}
**II-N**	2	-1.75	-1.45	-3.20
**II-O**	2	-19.30	16.11	-3.19
**II-F**	2	-14.93	10.28	-4.65
**II-S**	3	2.34	-5.15	-2.81
**II-Cl**	3	18.69	-24.15	-5.46
**II-Br**	4	21.72	-27.61	-5.89

As shown in Table S5, the RIAE relative ξE_{total}^{ae} total energies reproduce the same relative 60-DA stabilities obtained from the M06-2X/6-311G(d,p) gas-phase optimizations (see Table S2). For the halogenated MMs **II–X**, the 60-DA RIAE stabilization increases with the period of the halogen: –4.65 (**II–F,** period 2) < –5.46 (**II–Cl**, period 3) < –5.89 kcal·mol^{-1} (**II–Br**, period 4). In contrast, MMs **II-N**, **II-O,** and **II-S** show similar stabilization energies, ranging from –2.81 to –3.30 kcal·mol^{-1}.

Analysis of the relative ξE_{intra}^{A} intra-atomic and ξE_{inter}^{AB} interatomic energies contributing to the RIAE ξE_{total}^{ae} total energies for the series of acyclic MMs **II–X** shows that, except for MMs **II-N**, the other five MMs present the same dependence on the period of the X atom as that observed for the cyclic THPs **I-X** series (see Table 7).

A detailed analysis of the ξE_{intra}^{A} contributions to the ξE_{total}^{ae} total energies shows that, for the second-period systems **II-F** and **II-O**, the E_{intra}^{C2} intra-atomic energies at the anomeric C2 carbon, –11.72 (**II-F**) and –11.78 (**II-O**) kcal·mol^{-1}, are the principal source of 60-DA stabilization. This C2-centered stabilization was proposed by Popelier et al. for **II-O**.[21] IQA analysis further indicates that the dominant stabilizing term at 60-DA is the

favorable nuclei–electron interaction at the C2 carbon, –57.96 (**II-F**) and –61.08 kcal·mol^{-1} (**II-O**), as shown in Table S6.

Table S6. M06-2X/6-311G(d,p) gas-phase IQA E_{intra}^{A} intra-atomic energies, in a.u., and the relative E_{intra}^{C2} intra-atomic energies, in kcal·mol^{-1}, of the C2 carbon of the 60- and 180-DA conformations of substituted MMs **II-X**

	E_{intra}^{A}	T(A)	Vne(A)	Vee(A)
**II-F-a**	-37.0783	37.2004	-85.3524	11.0737
**II-F-e**	-37.0596	37.1815	-85.2601	11.0189
E_{intra}^{C2}	-11.72	11.88	-57.96	34.36
**II-O-a**	-37.1358	37.2485	-85.6527	11.2684
**II-O-e**	-37.1170	37.2309	-85.5553	11.2075
E_{intra}^{C2}	-11.78	11.07	-61.08	38.23

For **II–S**, **II–Cl**, and **II–Br**, which contain X atoms from periods higher than 2, the main contributions to ξE_{inter}^{AB} interatomic energies arise from the favorable O1–C2 interatomic interactions. The O1 and C2 interatomic energies ξE_{inter}^{AB} equal –4.58 and –2.82 (**II-S**), –17.56 and –9.82 (**II-Cl**) and –20.84 and –21.04 (**II-Br**) kcal·mol^{-1}. The most favorable interactions O1–C2 E_{inter}^{AB} interatomic energies are 15.43 (**II-S**), –36.93 (**II-Cl**) and –45.83 (**II-Br**) kcal·mol^{-1}, indicating that this interaction dominates the 60-DA stabilization in X-substituted species containing elements from periods higher than 2.

Finally, Figure S2 shows a graphical representation of the relative ξE_{intra}^{A} intra-atomic, ξE_{inter}^{AB} interatomic, and ξE_{total}^{ae} total energies between the 60- and 180-DA conformations of substituted MMs **II-X** given in Tables S5. Some appealing conclusions can be obtained from the analysis of graphical data shown in Figure S2: (i) least at MM **II-N**, both cyclic THP and acyclic MM series present a very similar energetic response to the nature of the X atom at the anomeric C2 carbon; (ii) the 60-DA stabilization in acyclic MM **II-X** is closely than the axial stabilization in cyclic THP **I-X**, indicating that the named *anomeric effect* is not limited to cyclic systems; (iii) the series of MM **II-X** derivatives can be divided into two groups: Group I, comprising the second-period derivatives **II–O** and **II–F**, and Group II, comprising **II–S**, **II–Cl**, and **II–Br**, in which the X atom belongs to periods higher than 2. In Group I, the *anomeric effect* is controlled by the favorable intra-atomic energies, $\xi E_{intra}^{A} < 0$, while in Group II, it is controlled by de favorable interatomic contributions, $\xi E_{inter}^{AB} < 0$. The sulfur S atom belonging to Group II shows a smaller axial stabilization; (iv) for the Cl- and Br-containing derivatives, the

magnitude of the *anomeric effect* increases with the period of the X atom due to markedly enhanced stabilizing ξE_{inter}^{AB} interatomic energies between the O1 and C2 atoms; and finally, (v) except for MM **II-N**, the ξE_{intra}^{A} intra-atomic and ξE_{inter}^{AB} interatomic energies contribute in opposite directions in the other five pairs of stereoisomers, with the stabilizing or destabilizing role of each term being correlated with the period of the X atom (see Figure S2).

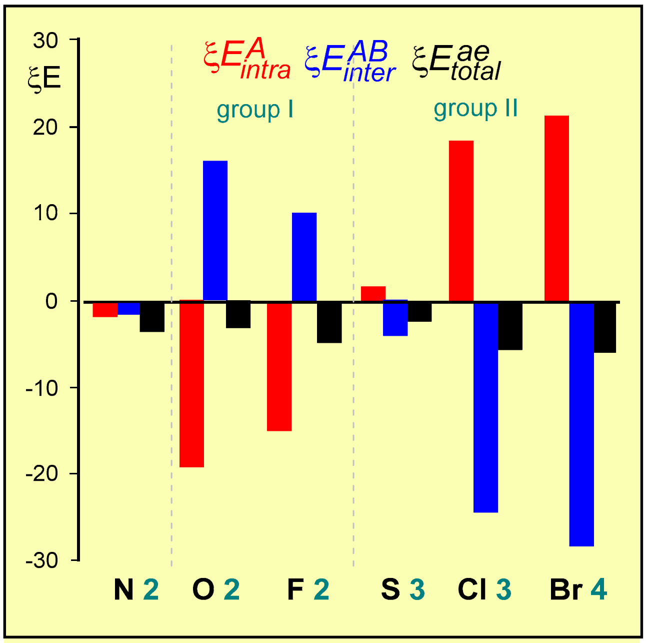

Figure S2. Graphical representation of the relative ξE_{intra}^{A} intra-atomic, in red, ξE_{inter}^{AB} interatomic, in blue, and ξE_{total}^{ae} total, in black, energies between the 60- and 180-DA conformations of substituted MMs **II-X**. ξE_{total}^{ae} total energies represent the RIAE relative energies between the two stereoisomers The period of the X atom is given in green. Energies are reported in kcal·mol^{-1}.

Table S7. M06-2X/6-311G(d,p) gas-phase total energies, in a.u, of the axial and equatorial conformations of cyclic 2-substituted THPs **I-X**, and the 60- and 180-DA conformations of acyclic substituted MMs **II-X**.

**I-N-a**	-405.659309	**II-N-a**	-288.935989
**I-N-e**	-405.654849	**II-N-e**	-288.930986
**I-O-a**	-386.235109	**II-O-a**	-269.507708
**I-O-e**	-386.228123	**II-O-e**	-269.502621
**I-F-a**	-370.967307	**II-F-a**	-254.238663
**I-F-e**	-370.962162	**II-F-e**	-254.231356
**I-S-a**	-709.206535	**II-S-a**	-592.482090
**I-S-e**	-709.202909	**II-S-e**	-592.477644
**I-Cl-a**	-731.324894	**II-Cl-a**	-614.597835
**I-Cl-e**	-731.318102	**II-Cl-e**	-614.589422
**I-Br-a**	-2845.298324	**II-Br-a**	-2728.571704
**I-Br-e**	-2845.290458	**II-Br-e**	-2728.562554

Table S8. M06-2X/6-311G(d,p) gas-phase total energies, in a.u, of the axial and equatorial conformations of substituted Chs **III-X**.

**III-N-a**	-369.751527
**III-N-e**	-369.749841
**III-O-a**	-350.323863
**III-O-e**	-350.319788
**III-F-a**	-335.058706
**III-F-e**	-335.058033
**III-S-a**	-673.303147
**III-S-e**	-673.303522
**III-Cl-a**	-695.422640
**III-Cl-e**	-695.422746
**III-Br-a**	-2809.395908
**III-Br-e**	-2809.395929
**III-Me-a**	-275.119997
**III-Me-e**	-275.122385
**III-tBu-a**	-393.025790
**III-tBu-e**	-393.033047

Table S9. M06-2X/6-311G(d,p) gas-phase relative axial/equatorial E_{intra}^{A} intra-atomic and E_{inter}^{AB} interatomic energies, in kcal·mol^{-1}, associated with the O1, C2 and X atoms of THPs **I-X**.

		E_{intra}^{A}	E_{inter}^{AB}
**I-N**	O1	-3.42	2.94
	C2	-9.25	6.78
	N	1.54	-4.51
**I-O**	O1	-1.54	0.77
	C2	-13.76	10.77
	O	4.18	-6.02
**I-F**	O1	3.72	-5.76
	C2	-15.33	11.07
	F	-5.42	5.78
**I-S**	O1	1.80	-2.47
	C2	4.67	-2.36
	S	-3.05	-2.89
**I-Cl**	O1	12.91	-16.06
	C2	9.68	-8.89
	Cl	-4.26	-2.43
**I-Br**	O1	17.39	-20.94
	C2	16.13	-12.14
	Br	-10.17	-0.97

M06-2X/6-311G(d,p) computed total energies and Cartesian coordinates of the axial and equatorial conformations of cyclic 2-substituted THPs **I-X**.

I-N-a
E(RM062X) = -405.659309 A.U.

C -2.74015000 -0.60229000 0.11158100
C -1.21590800 -0.49305500 0.17862400
O -0.83339200 0.86648300 0.10937600
C -1.35033900 1.65542500 1.17665400
C -2.87419000 1.63207300 1.20209400
C -3.37954400 0.18761000 1.25476300
H -0.76818700 -0.94861500 -0.72113500
H -3.03855500 -0.16387200 -0.84515700
H -3.06240600 -1.64450100 0.09892000
H -0.95441900 1.28929800 2.13062400
H -0.97259000 2.66289300 1.00031100
H -3.23401700 2.20821100 2.05873000
H -3.25063200 2.11512000 0.29427900
H -3.10101000 -0.26159200 2.21330700
H -4.46886500 0.15753900 1.17812700
C 0.79928200 -1.02190400 1.35588000
H 1.11049400 0.00761300 1.18735200
H 1.19962400 -1.36052600 2.31296000
H 1.22902400 -1.64855300 0.55497200
N -0.65581400 -1.09596100 1.38437800
C -1.08965100 -2.46739200 1.59004600
H -0.90614200 -3.10872900 0.70964900
H -0.53714400 -2.88791400 2.43158000
H -2.15063700 -2.51049300 1.83772300

I-N-a
E(RM062X) = -405.654849 A.U.

C -3.58669800 -0.06524400 0.52398100
C -2.65130400 0.15427000 -0.65946200
O -2.02804700 1.42302000 -0.60572900
C -1.22397200 1.59005900 0.54606600
C -2.09235200 1.47476600 1.80646800
C -2.81155700 0.12330500 1.82839200
H -3.18846400 0.12097500 -1.60752700
H -4.40017500 0.66501900 0.46906600
H -4.02924100 -1.06327700 0.46626600
H -0.46495500 0.78996400 0.56945000
H -1.46964500 1.59854900 2.69705100
H -2.83123000 2.28209200 1.79694200
H -2.07093100 -0.67961000 1.92701500
H -3.47562800 0.05195900 2.69259400
H -1.88053200 -0.63216700 -0.67440900
C -1.36313400 4.01103300 0.34000900

H -1.73828400 4.30708500 1.33360000
H -0.79389000 4.84932300 -0.06752000
H -2.20920700 3.81743800 -0.31815200
N -0.50929600 2.83538900 0.37623200
C 0.59222800 2.99227800 1.30333300
H 1.23100800 2.10654200 1.27682100
H 1.19440100 3.85118900 0.99913300
H 0.27294300 3.16076500 2.34610300

I-O-a
E(RM062X) = -386.235109 A.U.

C -2.83570700 -0.47433300 0.00639000
C -1.31579400 -0.41195000 0.04449000
O -0.84235800 0.91376800 0.09645200
C -1.33038200 1.64368700 1.21845600
C -2.85197900 1.70787000 1.22066100
C -3.42901800 0.29090900 1.19079200
H -0.86956300 -0.82407000 -0.86882500
H -3.16555500 -0.02418200 -0.93438300
H -3.14215100 -1.52186200 0.01182700
H -0.97480200 1.17634700 2.14534600
H -0.88538800 2.63522800 1.13657000
H -3.19389600 2.25462800 2.10334500
H -3.18321900 2.26147600 0.33587300
H -3.16880600 -0.22960000 2.11682900
H -4.51848800 0.31548300 1.12021400
O -0.89447900 -1.15484600 1.15664000
C 0.51138300 -1.25606500 1.25450500
H 0.97262000 -0.27565500 1.40271500
H 0.72620800 -1.89644300 2.10812500
H 0.93228100 -1.70837900 0.34812600

I-O-e
E(RM062X) = -386.228123 A.U.

C -2.91670800 -0.54253700 0.05121600
C -1.39718800 -0.41794400 0.04613300
O -0.98841800 0.93540400 -0.03219300
C -1.43693700 1.70024500 1.07095000
C -2.96293800 1.72435200 1.10766900
C -3.49618800 0.29153300 1.19530200
H -0.95416600 -0.92021500 -0.81377200
H -3.30270500 -0.17614100 -0.90517000
H -3.20107700 -1.59377400 0.14557200
H -1.05363600 1.24691100 1.99679400
H -3.28839300 2.32978700 1.95707900
H -3.33270100 2.19294600 0.19084300
H -3.19445600 -0.14968300 2.15259000
H -4.58767000 0.28489400 1.17126200

H -0.98388300 -0.87289900 0.95958200
O -0.84151200 2.95195700 1.01091000
C -1.16199100 3.72343300 -0.13364700
H -2.16730600 4.15147500 -0.06279300
H -0.43819100 4.53568300 -0.16712500
H -1.07851400 3.12717300 -1.04452700

I-F-a
E(RM062X) = -370.967307 A.U.

C -2.92286500 -0.43871600 0.00184200
C -1.40624900 -0.35618200 -0.00348500
O -0.92576800 0.93672100 0.02776500
C -1.39303300 1.70063100 1.14176600
C -2.91360400 1.76520900 1.17203100
C -3.48884700 0.34718000 1.18588000
F -0.94303400 -1.04405600 1.11616800
H -0.95625200 -0.83081000 -0.87572500
H -3.28142800 -0.01439300 -0.94065100
H -3.21757900 -1.48918000 0.03579400
H -1.00983600 1.25435300 2.06547200
H -0.94752600 2.68682200 1.02032900
H -3.23627000 2.33040900 2.04983000
H -3.26518900 2.29916400 0.28322400
H -3.20345400 -0.15363000 2.11573100
H -4.57940500 0.36532800 1.14479500

I-F-e
E(RM062X) = -370.962162 A.U.

C -2.91357400 -0.46142600 0.01215000
C -1.39166600 -0.38216100 0.04303000
O -0.94999600 0.96703000 0.02428500
C -1.39787000 1.67481400 1.13749700
C -2.90952200 1.75511500 1.17887400
C -3.49541100 0.33991700 1.17917000
H -0.94135200 -0.85622300 -0.82783300
H -3.26616100 -0.04412600 -0.93562600
H -3.22956500 -1.50663700 0.05680700
H -0.99054900 1.22235600 2.05569200
H -3.21932800 2.32262600 2.05864000
H -3.22383000 2.30258400 0.28570200
H -3.24028800 -0.15853600 2.12168600
H -4.58455100 0.37809000 1.12474200
F -0.86953900 2.92841000 1.02640000
H -1.00747700 -0.88236300 0.94504100

I-S-a
E(RM062X) = -709.206535 A.U.
C -2.81884800 -0.60529100 0.17232300

C -1.29609100 -0.49017200 0.21910200
O -0.86253400 0.84549200 0.17668200
C -1.41703500 1.68505100 1.18461600
C -2.93882000 1.69685200 1.12813500
C -3.47537600 0.26872000 1.24330400
H -0.84691100 -0.96002000 -0.65676300
H -3.13691400 -0.26110700 -0.81708800
H -3.11135500 -1.65269700 0.27094800
H -1.07977100 1.34424300 2.17110500
H -1.00041100 2.67398600 0.99492000
H -3.32810400 2.32993300 1.92946200
H -3.25464000 2.13316500 0.17485000
H -3.24887700 -0.12873600 2.23750100
H -4.56094000 0.25141600 1.12655000
C 1.11091800 -1.10235600 1.38369700
H 1.29045600 -0.03380100 1.27573000
H 1.66083400 -1.48178400 2.24318500
H 1.44027300 -1.62594900 0.48593300
S -0.65205600 -1.39797600 1.68815700

I-S-a
E(RM062X) = -709.202909 A.U.

C -2.92675000 -0.52720900 0.02073100
C -1.40684300 -0.45185600 0.08427500
O -0.95513000 0.89430900 0.10053600
C -1.42421600 1.60650400 1.22604100
C -2.94925600 1.66369900 1.23726700
C -3.52744000 0.24522500 1.19592700
H -0.94123300 -0.91227700 -0.78692500
H -3.26103500 -0.08524600 -0.92313400
H -3.24573000 -1.57265000 0.03103800
H -1.06742300 1.11971300 2.14598100
H -3.28383000 2.20428500 2.12469800
H -3.28474900 2.22030900 0.35674400
H -3.28240100 -0.27183500 2.13121100
H -4.61670100 0.27995900 1.12570300
H -1.04165100 -0.96872500 0.98464600
C -1.17275000 3.86100200 -0.36115700
H -2.20796300 4.19826900 -0.33731200
H -0.52917300 4.70465500 -0.60506500
H -1.03754800 3.07925500 -1.10662800
S -0.62276000 3.23521500 1.25209700

I-Cl-a
E(RM062X) = -731.324894 A.U.

C -2.92008400 -0.43999700 0.02245200
C -1.40178500 -0.34956000 -0.01358700
O -0.92017000 0.93341000 0.05241200

C -1.40266800 1.71291700 1.14916800
C -2.92288500 1.77538600 1.16618300
C -3.49860300 0.35812800 1.19009900
H -0.97724300 -0.80291600 -0.90464600
H -3.27324500 -0.01289200 -0.92289100
H -3.22204300 -1.48713500 0.05344800
H -1.02350000 1.28113700 2.08199100
H -0.95568800 2.69627200 1.01338500
H -3.25106100 2.34938200 2.03593000
H -3.26811500 2.30134900 0.27033700
H -3.23611000 -0.13153000 2.13243300
H -4.58791400 0.37817700 1.12510700
Cl -0.70908500 -1.38256100 1.36174000

I-Cl-e
E(RM062X) = -731.318102 A.U.

C -2.91500300 -0.46761800 0.01431000
C -1.39423900 -0.38755600 0.04062300
O -0.95379400 0.96639400 0.00962200
C -1.40004500 1.67213300 1.12725500
C -2.91498800 1.75780700 1.17187500
C -3.49317100 0.33769300 1.17985200
H -0.94409500 -0.86150800 -0.83025500
H -3.27011200 -0.05392000 -0.93405800
H -3.23075200 -1.51266100 0.06339700
H -1.00036900 1.22420200 2.04509900
H -3.22734500 2.32366600 2.05114400
H -3.24406600 2.29846600 0.28019500
H -3.23491600 -0.15453300 2.12463000
H -4.58268400 0.37607600 1.12738300
H -1.00288200 -0.87758800 0.94449000
Cl -0.67483200 3.30996000 1.03641800

I-Br-a
E(RM062X) = -2845.298324 A.U.

C -2.92410700 -0.43404400 0.01956800
C -1.40782800 -0.33628700 -0.02829300
O -0.92424800 0.93861200 0.04830500
C -1.40699400 1.71904400 1.14459500
C -2.92716500 1.77933000 1.16555200
C -3.50163500 0.36156500 1.18871000
H -0.97712000 -0.79642300 -0.91198700
H -3.28102300 -0.00403900 -0.92426100
H -3.22386000 -1.48138600 0.04717900
H -1.02441400 1.28301300 2.07417800
H -0.96021600 2.70223900 1.00788900
H -3.25221800 2.35093600 2.03795800
H -3.27610800 2.30779400 0.27266200

H -3.23593300 -0.13020300 2.12889800
H -4.59102000 0.38101800 1.12506000
Br -0.63550300 -1.49262900 1.45885900

I-Br-e
E(RM062X) = -2845.290458 A.U.

C -2.91601200 -0.47041600 0.01430100
C -1.39523400 -0.39074100 0.03950100
O -0.95496100 0.96430000 0.00866300
C -1.40331900 1.66739000 1.12663100
C -2.91792400 1.75777500 1.17047400
C -3.49367400 0.33557700 1.17950600
H -0.94587600 -0.86413200 -0.83226200
H -3.27202200 -0.05780300 -0.93424300
H -3.23166100 -1.51544200 0.06471100
H -1.00192800 1.22513500 2.04550900
H -3.23099400 2.32279600 2.04987700
H -3.25016300 2.29714000 0.27940100
H -3.23476500 -0.15537200 2.12474700
H -4.58342200 0.37327600 1.12724700
H -1.00291800 -0.88106200 0.94276700
Br -0.60131400 3.44897300 1.03924900

M06-2X/6-311G(d,p) computed total energies and Cartesian coordinates of the 60- and 180-DA conformations of acyclic substituted MMs **II-X**.

II-N-a
E(RM062X) = -288.935989 A.U.

C -1.13900100 -0.45746100 0.21528800
O -0.82301700 0.90131300 0.13899100
C -1.40959100 1.65837400 1.17803000
H -0.75158500 -0.90291700 -0.71564400
H -1.04475800 1.33851700 2.15723800
H -1.14245300 2.69906300 1.00231500
C 0.88297000 -1.09765800 1.31825700
H 1.24307000 -0.08764200 1.12364100
H 1.29732300 -1.44312600 2.26704800
H 1.24805800 -1.75967300 0.51347500
N -0.56930100 -1.08082200 1.39311400
C -1.10671500 -2.41329600 1.59250600
H -0.87352800 -3.09569400 0.75603600
H -0.68678800 -2.84251400 2.50382700
H -2.19152800 -2.36582900 1.70762500
H -2.22846100 -0.60173600 0.25402500
H -2.50270400 1.55923500 1.15946100

II-N-a
E(RM062X) = -288.930985 A.U.

C -0.00033500 0.00165400 0.00003400
O -0.00027200 0.00353000 1.40476900
C 1.29939300 -0.00163900 1.92967600
N 1.22102500 -0.12544000 3.35941000
C 0.65420800 -1.40879400 3.74305700
H -1.03921700 0.02029100 -0.32439500
H 1.81689200 0.93728700 1.68566900
H 0.51386100 0.88474300 -0.40159200
H 1.30078700 -2.25249500 3.44094200
H 0.52703400 -1.44024800 4.82633500
H -0.31965900 -1.52624100 3.27154800
C 2.52562400 0.07627300 3.96115100
H 2.90682600 1.06668600 3.70431300
H 2.43499500 0.01834700 5.04687200
H 3.26575000 -0.67851700 3.63960700
H 0.48496600 -0.89787800 -0.40089700
H 1.87305300 -0.84130200 1.47524600

II-O-a
E(RM062X) = -269.507708 A.U.

C -1.26185500 -0.37553500 0.08964900
O -0.79947600 0.94018100 0.13451000

C -1.33582000 1.66680100 1.22249700
H -0.87586100 -0.79101100 -0.84804700
H -1.05185300 1.21804000 2.17848700
H -0.93783000 2.67773300 1.15928500
O -0.84848600 -1.13912500 1.18185300
C 0.55345100 -1.31984400 1.21800100
H 1.07515000 -0.36306800 1.30948300
H 0.77355900 -1.94190000 2.08344400
H 0.90280400 -1.82701900 0.31000400
H -2.35713000 -0.40714900 0.10645800
H -2.43061400 1.70479300 1.16168700

I-IO-e
E(RM062X) = -269.502621 A.U.

C -1.43059200 -0.35056500 0.02627000
O -0.82454300 0.91723500 0.02978100
C -1.28759800 1.71586900 1.08840800
H -1.01031600 -0.91101300 -0.80654300
H -0.97116500 1.29806800 2.05141300
H -1.22848200 -0.89424500 0.95839400
O -0.73702900 2.97528100 0.99742700
C -1.15131400 3.67365700 -0.16108600
H -2.24645700 3.73509700 -0.20391100
H -0.73853700 4.67794000 -0.08931900
H -0.78423900 3.18634700 -1.06640200
H -2.51818100 -0.27562800 -0.10537600
H -2.39260600 1.76249600 1.04731300

II-F-a
E(RM062X) = -254.238663 A.U.

C -0.00149900 0.01886400 0.00879900
O -0.00458100 0.01023000 1.37605300
C 1.29858400 -0.00378900 1.93436500
F 0.52450300 1.20254100 -0.47439000
H -1.03626500 -0.05143500 -0.32265900
H 1.84440100 0.90434400 1.67051300
H 1.18014500 -0.05986500 3.01379500
H 0.61966400 -0.78632400 -0.40395300
H 1.85812000 -0.87918200 1.58363300

II-F-e
E(RM062X) = -254.231356 A.U.

C 0.00300000 0.01943700 0.02925600
O -0.00978700 0.01781200 1.43792200
C 1.27252600 0.01854900 1.95741200
H -1.03310800 0.01934500 -0.30045700

H 1.81842100 0.92368100 1.65707200
F 1.15993900 0.01616600 3.31068900
H 0.50444100 0.91222500 -0.36513500
H 0.50530500 -0.87195600 -0.36717200
H 1.82067300 -0.88421300 1.65406400

II-S-a
E(RM062X) = -592.482089 A.U.

C -1.22234500 -0.43165800 0.25754200
O -0.82193400 0.90294700 0.22103500
C -1.47377700 1.71699100 1.17407000
H -0.83190700 -0.89877600 -0.64621900
H -1.23898200 1.40040800 2.19457400
H -1.12046400 2.73406200 1.01616800
C 1.15897700 -1.18595900 1.35787200
H 1.38785600 -0.13807600 1.16978900
H 1.70497500 -1.52538400 2.23614500
H 1.44519700 -1.79206700 0.49822600
S -0.61330500 -1.35999800 1.70806400
H -2.56116300 1.68532700 1.03182700
H -2.31426800 -0.52855500 0.27854300

II-S-a
E(RM062X) = -592.477644 A.U.

C -0.00212200 0.01875600 -0.01190600
O -0.01568100 0.03406700 1.39700600
C 1.28174300 -0.00596300 1.92861500
S 1.21354600 -0.08076300 3.72617100
C 0.36742800 -1.67867400 3.86856500
H -1.03812800 0.04947400 -0.34423100
H 1.84592800 0.89979200 1.67455200
H 0.52977800 0.89019400 -0.41405700
H 0.99744400 -2.48611800 3.49397500
H 0.16238100 -1.84443100 4.92480800
H -0.56928000 -1.64047500 3.31647400
H 0.47325300 -0.89262500 -0.39678100
H 1.82494500 -0.88029000 1.54423600

II-Cl-a
E(RM062X) = -614.597835 A.U.

C -0.00604600 0.01312700 0.02213200
O 0.00847900 0.04660800 1.38360600
C 1.30523100 -0.01404500 1.95224200
H -1.03403900 -0.08255700 -0.31069800
H 1.87430100 0.88820100 1.71489600
H 1.17186000 -0.09057900 3.02847400
H 0.63641000 -0.76751900 -0.39195100

H 1.84633700 -0.89360300 1.58526700
Cl 0.61059200 1.56558000 -0.73158900

II-Cl-e
E(RM062X) = -614.589422 A.U.

C 0.00136000 0.01957600 0.01856900
O -0.02254900 0.01776300 1.43133700
C 1.26221000 0.01834700 1.95046600
H -1.03402100 0.01899300 -0.31367600
H 1.81554200 0.91797300 1.66544800
H 0.50486200 0.91351600 -0.36890100
H 0.50659600 -0.87239900 -0.37116500
H 1.81737700 -0.87934600 1.66293200
Cl 1.15408400 0.01575200 3.72812500

II-Br-a
E(RM062X) = -2728.571704 A.U.

C -0.01506900 -0.00983300 0.03668500
O 0.00841700 0.03348500 1.39296600
C 1.30600600 -0.02121100 1.95983600
H -1.04196300 -0.10047500 -0.29831500
H 1.86903800 0.88274000 1.71326200
H 1.17407700 -0.09215100 3.03653200
H 0.63568600 -0.77699400 -0.38695900
H 1.84873500 -0.90045900 1.59533800
Br 0.64111600 1.68452100 -0.81441200

II-Br-e
E(RM062X) = -2728.562554 A.U.

C -0.00353000 0.01960300 0.00087600
O -0.02000800 0.01782100 1.41510600
C 1.26639400 0.01839300 1.92692600
H -1.04106200 0.01899600 -0.32504000
H 1.82140300 0.91878600 1.65073100
H 0.49803500 0.91365500 -0.38835700
H 0.49978000 -0.87249200 -0.39058700
H 1.82323500 -0.88006500 1.64812500
Br 1.16121500 0.01547900 3.86535100

M06-2X/6-311G(d,p) computed total energies and Cartesian coordinates of the axial and equatorial conformations of substituted CHs **III-X**.

III-N-a
E(RM062X) = -369.751526 A.U.

C -2.76682500 -0.57217000 0.11159700
C -1.23075700 -0.52902300 0.12096700
C -1.34106000 1.76991100 1.16697000
C -2.86849400 1.67650800 1.24503300
C -3.34431900 0.22047600 1.28732100
H -0.85210400 -1.08617800 -0.75782600
H -3.10302000 -0.12396500 -0.82976200
H -3.13728900 -1.59871700 0.10769800
H -0.90818500 1.39137300 2.09809200
H -1.03326900 2.81363400 1.06113400
H -3.23716400 2.22082200 2.11843100
H -3.29905900 2.16439100 0.36149900
H -3.01609800 -0.23961900 2.22444500
H -4.43670400 0.18135900 1.26595200
C 0.76126600 -1.05464500 1.39798800
H 1.09166500 -0.02143700 1.50556100
H 1.12005400 -1.60366200 2.26965400
H 1.24073400 -1.48333600 0.49940500
N -0.69024300 -1.13342500 1.34702200
C -0.80764600 0.94288700 -0.00584300
H 0.27553100 1.04016700 -0.09720700
H -1.22731700 1.32641600 -0.94224900
C -1.11647800 -2.51517900 1.50488100
H -2.18906400 -2.57324000 1.69037100
H -0.88056300 -3.13479900 0.62083500
H -0.61260700 -2.94986100 2.36927400

III-N-e
E(RM062X) = -369.749840 A.U.

C -3.62294200 -0.09699800 0.59941400
C -2.74569800 -0.05421400 -0.64892600
C -1.14191100 1.57741700 0.48162300
C -2.01320800 1.48181800 1.74166400
C -2.76845300 0.15439200 1.83886700
H -3.34578000 -0.20835800 -1.54932400
H -4.39531900 0.67790100 0.52732100
H -4.13768500 -1.05832400 0.67533800
H -0.34757100 0.80410900 0.55375000
H -1.40133800 1.59872200 2.63738200
H -2.72274000 2.31803000 1.71964800
H -2.04670000 -0.66435500 1.94698600
H -3.38499700 0.15129200 2.74134000
H -2.02340900 -0.87846500 -0.60613000

C 0.28115600 3.22967100 1.54711400
H 1.04274100 2.45844000 1.76373100
H 0.79590600 4.17589400 1.37567600
H -0.34420300 3.35712500 2.42919800
N -0.52203600 2.90605300 0.37580400
C -1.99029900 1.27197900 -0.76048000
H -1.36204100 1.23894600 -1.65185800
H -2.69880200 2.09912900 -0.89108600
C 0.30320000 3.03126500 -0.81810700
H -0.30557500 3.01089100 -1.72052500
H 0.81754800 3.99284100 -0.79618900
H 1.06636900 2.23500400 -0.88983000

III-O-a
E(RM062X) = -350.323863 A.U.

C -2.87168300 -0.49756500 0.02928900
C -1.34828700 -0.43204900 -0.01846300
C -1.39510200 1.78515400 1.17998300
C -2.92426100 1.72133500 1.23809800
C -3.41660300 0.27145700 1.23641200
H -0.97692000 -0.95323300 -0.91384400
H -3.26878700 -0.07057600 -0.89817200
H -3.17653800 -1.54574000 0.07091700
H -0.98175300 1.33321600 2.08795500
H -1.05620600 2.82377400 1.15091900
H -3.29176700 2.24390700 2.12504700
H -3.33669100 2.24464100 0.36621700
H -3.07591900 -0.22746600 2.14806600
H -4.50912500 0.24054500 1.23604200
O -0.89139500 -1.12832300 1.13205400
C 0.50390400 -1.27900100 1.20431300
H 1.00860700 -0.34181600 1.46734300
H 0.70833600 -2.01391000 1.98222200
H 0.91440900 -1.64396200 0.25328400
C -0.87011500 1.02404200 -0.03924500
H 0.22082300 1.07049300 -0.09230700
H -1.24585700 1.48951700 -0.95737200

III-O-e
E(RM062X) = -350.319788 A.U.

C -2.98599600 -0.59592100 0.09471100
C -1.45971500 -0.56469200 -0.02590900
C -1.42395800 1.73323100 0.98339700
C -2.95085900 1.73214400 1.07102700
C -3.46844800 0.29980500 1.23917900
H -1.13168500 -1.17804400 -0.86860900
H -3.42667000 -0.24357500 -0.84567800
H -3.33362300 -1.62138400 0.24367700

H -1.03664300 1.28438200 1.90517800
H -3.26362600 2.36753700 1.90396000
H -3.37692800 2.15503200 0.15403100
H -3.10457900 -0.10503300 2.19084200
H -4.55986700 0.29733900 1.29110700
H -1.01837300 -1.00380300 0.87650700
O -0.85828600 3.03406000 0.99136600
C -1.19310900 3.86736000 -0.09294200
H -2.26812500 4.07348600 -0.14252000
H -0.67006200 4.80899000 0.06958100
H -0.86702800 3.45257300 -1.05320800
C -0.95086400 0.87133600 -0.18808600
H 0.14085200 0.90181900 -0.23793000
H -1.33703600 1.27717200 -1.12992000

III-F-a
E(RM062X) = -335.058706 A.U.

C -2.98476600 -0.45917300 0.01098100
C -1.47073400 -0.38946700 -0.11289900
C -1.44547700 1.81279200 1.10179700
C -2.97261000 1.76573100 1.20140000
C -3.47125400 0.31865900 1.23650000
F -0.92073400 -1.03637100 0.99998200
H -1.12362600 -0.93965800 -0.99167200
H -3.42311000 -0.03756800 -0.90056000
H -3.28868000 -1.50709500 0.06743900
H -1.01147900 1.36138500 1.99920600
H -1.09357200 2.84604300 1.05729300
H -3.30949000 2.30300100 2.09128000
H -3.40843200 2.27833100 0.33469300
H -3.08980200 -0.17265100 2.13680500
H -4.56200600 0.28789900 1.28891400
C -0.95109300 1.03939600 -0.12346500
H 0.14080800 1.02008900 -0.15821600
H -1.30125900 1.52468100 -1.04135000

III-F-e
E(RM062X) = -335.058033 A.U.

C -2.94800700 -0.47513700 0.01399600
C -1.41807600 -0.44515700 -0.04341500
C -1.42372400 1.76618500 1.13117200
C -2.94015000 1.76065400 1.18663300
C -3.46784800 0.32283400 1.21293200
H -1.05619700 -0.98578000 -0.92063700
H -3.35044600 -0.04038100 -0.90836500
H -3.30475300 -1.50682300 0.06302600
H -1.01321700 1.34691500 2.05803800
H -3.27458400 2.32360500 2.06095800

H -3.31207500 2.28107500 0.29709700
H -3.14154300 -0.16614900 2.13850900
H -4.55987300 0.32667700 1.22734100
F -0.97732600 3.08269700 1.05404100
H -1.01546900 -0.96315500 0.83510100
C -0.89724300 0.99514300 -0.06493900
H 0.19478100 1.02355800 -0.06439000
H -1.24003400 1.50487800 -0.97213300

III-S-a
E(RM062X) = -673.303146 A.U.

C -2.78133100 -0.62287700 0.19796500
C -1.25248800 -0.50533500 0.21394200
C -1.48037400 1.84356500 1.16778400
C -3.00521900 1.71862800 1.11475800
C -3.44511200 0.25934300 1.26014500
H -0.84048100 -1.07196200 -0.62585300
H -3.12550100 -0.30552100 -0.79360500
H -3.07515000 -1.66810300 0.32153900
H -1.12651400 1.53910500 2.15783000
H -1.17865200 2.88379200 1.02190900
H -3.46268200 2.33064900 1.89606500
H -3.36451700 2.10731800 0.15363400
H -3.17719800 -0.10256100 2.25808500
H -4.53165300 0.17946800 1.17391800
C 1.15496400 -1.24384200 1.41776500
H 1.54002500 -0.23314000 1.54955400
H 1.63669000 -1.89946600 2.14195600
H 1.38458900 -1.60277600 0.41333200
S -0.62794400 -1.33314600 1.72932300
C -0.82347500 0.96210200 0.10386900
H 0.26519700 1.04780800 0.14595800
H -1.12450200 1.31606700 -0.89049400

III-S-e
E(RM062X) = -673.303522 A.U.

C -2.97239400 -0.59516000 0.07317000
C -1.44252800 -0.61576200 0.07537600
C -1.40862700 1.64956100 1.18298400
C -2.93847000 1.67488100 1.18453100
C -3.50839200 0.25260200 1.22843700
H -1.06602600 -1.19707000 -0.77013200
H -3.32464400 -0.17337700 -0.87593600
H -3.36608800 -1.61285100 0.13436200
H -1.07031800 1.20138200 2.12460200
H -3.29604600 2.26360400 2.03204200
H -3.29190100 2.16980400 0.27239600
H -3.22881200 -0.21675100 2.17917400

H -4.60042700 0.28896900 1.20259600
H -1.09010700 -1.11471000 0.98594700
C -1.15959000 4.03307800 -0.32262800
H -2.24346500 4.06769600 -0.42668400
H -0.78068000 5.05450800 -0.32308100
H -0.71613500 3.49941300 -1.16223300
S -0.66081100 3.31717100 1.26786600
C -0.86966100 0.80503800 0.02710500
H 0.22201300 0.78662100 0.06102200
H -1.15881700 1.26945300 -0.92289100

III-Cl-a
E(RM062X) = -695.422640 A.U.

C -2.98012000 -0.46225700 0.03250600
C -1.46296200 -0.40133600 -0.10993500
C -1.45112200 1.82071300 1.11769500
C -2.97950900 1.77629000 1.19657200
C -3.48460800 0.33167300 1.23905100
H -1.14159500 -0.91431700 -1.01489600
H -3.40041100 -0.03211800 -0.88530500
H -3.30426100 -1.50345900 0.08147600
H -1.02631800 1.39636800 2.03271200
H -1.10090900 2.85336300 1.05143200
H -3.32782400 2.32377000 2.07551200
H -3.40155800 2.28089900 0.31855900
H -3.13662500 -0.14953500 2.15830400
H -4.57659600 0.30835900 1.25931500
Cl -0.68263400 -1.35928600 1.23005800
C -0.94008400 1.03093200 -0.08883400
H 0.15100400 1.02568600 -0.12338700
H -1.29365500 1.50910500 -1.01090900

III-Cl-e
E(RM062X) = -695.422746 A.U.

C -2.95903600 -0.48041300 0.01837700
C -1.43119800 -0.44662600 -0.05306900
C -1.42819700 1.76797400 1.11313000
C -2.94793400 1.76211700 1.18352700
C -3.46633900 0.31967100 1.22015500
H -1.07453500 -0.98705500 -0.93294200
H -3.37158300 -0.04857100 -0.90087600
H -3.31382100 -1.51239300 0.07326400
H -1.00743100 1.35587100 2.03261600
H -3.28191600 2.32059200 2.06024300
H -3.33889400 2.27560700 0.29851600
H -3.12920500 -0.16329500 2.14476600
H -4.55841900 0.32450000 1.24613100
H -1.01563000 -0.95898100 0.82266100

Cl -0.82043200 3.47421300 1.05525500
C -0.91142600 0.99490600 -0.09143700
H 0.17996800 1.01687900 -0.10585100
H -1.26253400 1.49361100 -1.00117100

III-Br-a
E(RM062X) = -2809.395908 A.U.

C -2.98320900 -0.45890400 0.03128600
C -1.46712200 -0.39641400 -0.11783100
C -1.45347400 1.82415800 1.11686400
C -2.98186600 1.77923800 1.19681400
C -3.48922400 0.33522900 1.23684300
H -1.14367300 -0.91188800 -1.01962100
H -3.40158000 -0.02350700 -0.88600500
H -3.31295400 -1.49821500 0.07530600
H -1.02721000 1.39971000 2.03106200
H -1.10441900 2.85717100 1.04962800
H -3.32914500 2.32523400 2.07707100
H -3.40439900 2.28614600 0.32036200
H -3.14506700 -0.14796500 2.15649100
H -4.58138800 0.31447800 1.25312200
Br -0.60352400 -1.45908600 1.32033300
C -0.94284000 1.03473700 -0.08959100
H 0.14772900 1.03488100 -0.12929400
H -1.30194900 1.51355800 -1.01020200

III-Br-e
E(RM062X) = -2809.395929 A.U.

C -2.96386600 -0.48226500 0.01836400
C -1.43590400 -0.44980700 -0.05559200
C -1.42985800 1.76302200 1.10983000
C -2.94979600 1.76093800 1.18422300
C -3.46751800 0.31703400 1.22186200
H -1.08110100 -0.99052000 -0.93614300
H -3.37772500 -0.04913000 -0.89969000
H -3.31929000 -1.51403300 0.07297600
H -1.00312900 1.36052500 2.02992600
H -3.28328200 2.31776600 2.06178400
H -3.34503300 2.27318600 0.30064000
H -3.12779200 -0.16602800 2.14547200
H -4.55962900 0.32285200 1.25100700
H -1.01981100 -0.96156800 0.82008000
Br -0.76329300 3.61910800 1.05536600
C -0.91348400 0.99220600 -0.09630700
H 0.17773100 1.01091600 -0.11435600
H -1.26556000 1.48838100 -1.00681500

III-Me-a

E(RM062X) = -275.119997 A.U.

C -2.96924700 -0.44660300 0.07124200
C -1.43217200 -0.42640400 0.04115500
C -1.49850400 1.87676600 1.14056700
C -3.02906400 1.84023400 1.13559400
C -3.54594000 0.40119500 1.20984800
H -1.10978800 -0.91735100 -0.88353000
H -3.34448400 -0.05142900 -0.88035500
H -3.32484400 -1.47884300 0.14749200
H -1.13009400 1.50939200 2.10471900
H -1.14415300 2.90658500 1.04528200
H -3.42801700 2.43263000 1.96325200
H -3.39296900 2.30176100 0.20915300
H -3.27065400 -0.03330400 2.17708500
H -4.63812900 0.38840600 1.16381600
C -0.92738300 1.02514900 0.00212400
C -0.82696500 -1.21228000 1.20752500
H -0.23370500 -2.05333400 0.83977100
H -0.17165300 -0.57373700 1.80532000
H -1.59151800 -1.61576400 1.87472800
H 0.16667400 1.03723500 0.03000800
H -1.22555100 1.47546300 -0.95211300

III-Me-e
E(RM062X) = -275.122385 A.U.

C -2.97184700 -0.47300900 0.00855300
C -1.44386600 -0.43918600 -0.07280700
C -1.41586300 1.78928300 1.14285700
C -2.94574600 1.73958300 1.22143900
C -3.47193700 0.30262500 1.22942800
H -1.09842800 -0.96821600 -0.96496000
H -3.38904800 -0.01993800 -0.89905100
H -3.32886000 -1.50571000 0.04119200
H -1.01879400 1.29691400 2.04143700
H -3.28902300 2.27696400 2.11100800
H -3.35685300 2.27085800 0.35239300
H -3.12671000 -0.20110200 2.14039900
H -4.56469500 0.29993400 1.26080000
H -1.02686200 -0.96903200 0.79214900
C -0.92531000 1.00046600 -0.07612000
C -0.90797800 3.22791800 1.11319000
H -1.23997000 3.78408900 1.99340500
H 0.18390000 3.26218700 1.08183000
H -1.28542700 3.74667800 0.22648800
H 0.16866400 1.01188700 -0.10922200
H -1.27507400 1.50933000 -0.98439300

III-tBu-a

E(RM062X) = -393.025790 A.U.

C -3.03293400 -0.41480900 0.28725000
C -1.48957700 -0.44584500 0.22709700
C -1.52377200 2.02353800 0.99573600
C -3.05059900 2.04835400 0.91781100
C -3.63168000 0.66303700 1.20484400
H -1.22857300 -0.98162400 -0.69437400
H -3.39653800 -0.22027600 -0.72875400
H -3.42620600 -1.39687700 0.56512300
H -1.20704700 1.79573300 2.01884200
H -1.11754300 3.01030600 0.75651700
H -3.46361000 2.78237400 1.61471800
H -3.34552800 2.36453900 -0.09049300
H -3.45529100 0.41963000 2.25468400
H -4.71700100 0.67781500 1.06938500
C -0.96604900 0.98943900 0.01605200
C -0.80778500 -1.29449600 1.35143300
C -1.39288700 -1.09438800 2.75685700
H -2.43737100 -1.41075100 2.80535500
H -0.83252400 -1.70611800 3.47001500
H -1.33044900 -0.05786900 3.09197800
C -0.98012000 -2.77903500 0.98806000
H -0.46962100 -3.01158500 0.04938900
H -0.55648800 -3.41607300 1.76941300
H -2.03331600 -3.04809200 0.87592100
C 0.69680500 -1.00191000 1.41050300
H 1.19184900 -1.72529800 2.06413900
H 1.15457700 -1.07623300 0.41938900
H 0.89941000 -0.00358200 1.80657800
H 0.12479600 1.00780500 0.01127600
H -1.27597000 1.29563900 -0.99117600

III-tBu-e
E(RM062X) = -393.033047 A.U.

C -2.96303300 -0.53626300 0.02403600
C -1.43542700 -0.49671300 0.00229100
C -1.44032200 1.79340400 1.10352500
C -2.97704400 1.74684700 1.09244700
C -3.50405600 0.31174100 1.17475800
H -1.05045400 -1.07131600 -0.84446900
H -3.34724800 -0.14065200 -0.92405400
H -3.31770500 -1.56711100 0.10630200
H -1.10669800 1.30529600 2.03267300
H -3.38927600 2.33500400 1.91553700
H -3.34278300 2.19802400 0.16207800
H -3.19386000 -0.13307800 2.12797400
H -4.59735100 0.31379800 1.16651000
H -1.05270000 -0.97720400 0.91098400

C -0.90696900 0.94017900 -0.05893800
C -0.86187300 3.23711800 1.15111500
C -1.39762000 4.11147300 0.00994400
H -2.47197700 4.28446300 0.10450000
H -0.90371400 5.08718400 0.02605900
H -1.20729900 3.66119500 -0.96772100
C 0.67059400 3.19439300 1.05975200
H 1.08678900 4.18101800 1.28006200
H 1.08760000 2.48395500 1.78062000
H 1.01214400 2.90931300 0.06270800
C -1.22818000 3.89438200 2.48995800
H -0.85455300 3.30090600 3.32977600
H -0.77982000 4.88950900 2.55627100
H -2.30649200 4.01383800 2.60931600
H 0.18442100 0.91607800 -0.04790600
H -1.20890000 1.39103800 -1.01269200
